# Synthesis of DOTA-pyridine chelates for ^64^Cu coordination and radiolabeling of αMSH peptide

**DOI:** 10.1186/s41181-020-00119-4

**Published:** 2021-01-13

**Authors:** Hua Yang, Feng Gao, Brooke McNeil, Chengcheng Zhang, Zheliang Yuan, Stefan Zeisler, Joel Kumlin, Jutta Zeisler, François Bénard, Caterina Ramogida, Paul Schaffer

**Affiliations:** 1 Life Sciences Division, TRIUMF, 4004 Wesbrook Mall, Vancouver, BC V6T 2A3 Canada; 2 Department of Chemistry, Simon Fraser University, 8888 University Dr, Burnaby, BC V5A 1S6 Canada; 3 Department of Molecular Oncology, BC Cancer Research Centre, 675 West 10th Ave, Vancouver, BC V5Z 1L3 Canada; 4 Department of Radiology, University of British Columbia, 2775 Laurel St, Vancouver, BC V5Z 1M9 Canada

**Keywords:** Copper-64, Chelating ligands, Radiolabeling, α-Melanocyte-stimulating hormone, Pyridyl, DOTA

## Abstract

**Background:**

^64^Cu is one of the few radioisotopes that can be used for both imaging and therapy, enabling theranostics with identical chemical composition. Development of stable chelators is essential to harness the potential of this isotope, challenged by the presence of endogenous copper chelators. Pyridyl type chelators show good coordination ability with copper, prompting the present study of a series of chelates DOTA-xPy (x = 1–4) that sequentially substitute carboxyl moieties with pyridyl moieties on a DOTA backbone.

**Results:**

We found that the presence of pyridyl groups significantly increases ^64^Cu labeling conversion yield, with DOTA-2Py, −3Py and -4Py quantitatively complexing ^64^Cu at room temperature within 5 min (1 × 10^− 4^ M). [^64^Cu]Cu-DOTA-xPy (x = 2–4) exhibited good stability in human serum up to 24 h. When challenged with 1000 eq. of NOTA, no transmetallation was observed for all three ^64^Cu complexes. DOTA-xPy (x = 1–3) were conjugated to a cyclized α-melanocyte-stimulating hormone (αMSH) peptide by using one of the pendant carboxyl groups as a bifunctional handle. [^64^Cu]Cu-DOTA-xPy-αMSH retained good serum stability (> 96% in 24 h) and showed high binding affinity (Ki = 2.1–3.7 nM) towards the melanocortin 1 receptor.

**Conclusion:**

DOTA-xPy (x = 1–3) are promising chelators for ^64^Cu. Further in vivo evaluation is necessary to assess the full potential of these chelators as a tool to enable further theranostic radiopharmaceutical development.

**Supplementary Information:**

The online version contains supplementary material available at 10.1186/s41181-020-00119-4.

## Background

Personalized medicine is predicated on a philosophy of tailoring disease diagnosis, monitoring and therapy for individual patients in order to ensure maximal medical benefit during disease management (Vogenberg et al. 2010). Theranostics is an approach in which a single compound can be used to both diagnose and treat disease, and is an important tool in the move toward personalized medicine (Yordanova et al. 2017). Quantitative target visualization of a therapeutic radiopharmaceutical can help predict if a patient will benefit from a therapy and avoid unnecessary or ineffective treatments. In many instances, modern radiotheranostics include isotopes used for therapy, such as yttrium (^90^Y), lutetium-177 (^177^Lu) and actinium-225 (^225^Ac), and pairs them with compounds containing a different, imageable isotope for imaging, such as gallium-68 (^68^Ga), fluorine-18 (^18^F) and indium-111 (^111^In) (Langbein et al. 2019; Yordanova et al. 2017). One key assumption is that the imaging tracer has identical biodistribution as the therapeutic radiopharmaceutical. This assumption is not always true considering differences in molar activity (activity per mole of compound), in vivo stability and inherent biological sensitivity to even the slightest perturbations in pharmaceutical structure (Di 2015). The above matched-pair philosophy is driven primarily by the fact that not many isotopes are capable of both imaging and therapy. Copper-64 (^64^Cu, t_1/2_ = 12.7 h) decays via electron capture (branching ratio [BR] = 44%), β^−^ emission (39%, 0.573 MeV), and β^+^ emission (17%, E_β,avg_ = 0.655 MeV). Its half-life makes the radionuclide a versatile isotope for imaging, therapy or theranostics using small molecules, peptides, antibodies, and nanoparticle targeting and carrier platforms (Cai and Anderson 2014; Gutfilen et al. 2018). While the unique decay profile permits ^64^Cu, in principle, to be a dual PET-imaging and targeted therapy isotope, the high positron branching ratio precludes the clinical use of ^64^Cu for radiotherapy. The pure β^−^ emitter, ^67^Cu (t_1/2_ = 61.8 h) is available by high gamma irradiation and shows promise as an element-matched theranostic pair for ^64^Cu PET imaging and ^67^Cu targeted therapy (Smith et al. 2012). As one of the only copper isotopes that can be produced via proton irradiation on lower energy medical cyclotrons, ^64^Cu is more readily available over other isotopes, making it more ideal for use in imaging (McCarthy et al. 1997).

**Fig. 1:**
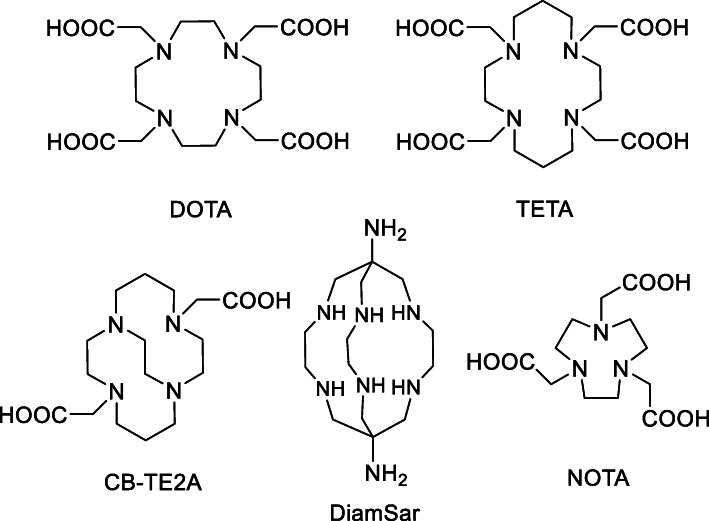
Examples of commonly used ^64^Cu chelators

The development of chelators for Cu is essential in order to harness its theranostic power. Cu is the third most abundant metal in the human body, and plays an important role in many biological functions, such as redox electron transfer, structure shaping, and catalysis (Gutfilen et al. 2018). As a result, there are many Cu chelating proteins in vivo and it is a challenge to develop thermodynamically and kinetically stable Cu chelators. DOTA (1,4,7,10-tetraazacyclododecane- 1,4,7,10-tetraacetic acid) (De León-Rodríguez and Kovacs 2008), TETA (1,4,8,11-tetraazacyclote- tradecane-1,4,8,11-tetraacetic acid) (Wadas and Anderson 2007) and their derivatives are commonly used, but they are unstable in vivo (Fig. 1) (Smith 2004). Over time, NOTA (1,4,7-triazacyclononane-1,4,7-triacetic acid) (Cooper et al. 2012), the cross-bridged cyclam (CB-TE2A) (Sprague et al. 2007), and sarcophagine-based chelators (Di Bartolo et al. 2006) have been developed and show improved stability (Anderson and Ferdani 2009; Cai and Anderson 2014).

**Scheme 1:**
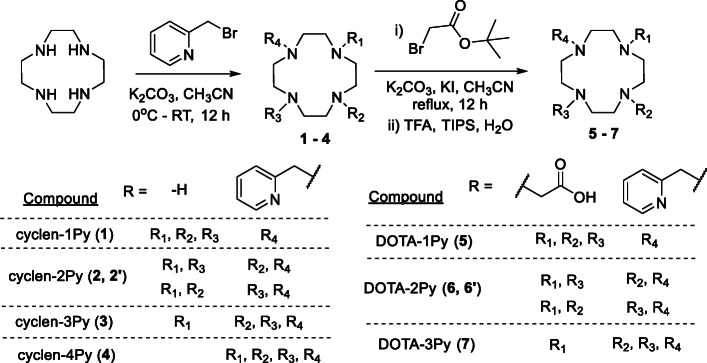
Synthesis of cyclen-xPy (x = 1–4, compounds **1**–**4**) and DOTA pyridine derivatives DOTA-xPy (x = 1–3, compounds **5**–**7**).

The development of bifunctional chelators capable of complexing radiometals at ambient temperature, in short reaction times, and sub-micromolar concentrations is of increasing interest. Chelators containing pyridyl moieties, including pyridine, bipyridine and terpyridine, have been previously used to complex Cu (Czerwińska et al. 2017; Lee et al. 2011; Leussing and Hansen 1957), and as Cu(II) is a d9 metal of borderline hardness, it favors ligation with softer bases including amines and imines. We hypothesized for this study that the addition of pyridine moieties in place of the carboxylic acid prostheses on DOTA (referred to herein as DOTA-xPy, x = 1–4; compounds 4–8 in Scheme 1) would maintain a higher affinity for the Cu(II) ion.

Furthermore, we present the application of novel DOTA-pyridine derivatives and the labeling of a peptide targeting the Melanocortin 1 receptor (MC1R) (referred to herein as DOTA-xPy-αMSH (x = 1–3)). MC1R is overexpressed in primary and metastatic melanoma (López et al. 2007; Tafreshi et al. 2019). With low normal tissue expression, this receptor is an attractive target for molecular imaging and treatment of late-stage melanoma, which has a low long-term survival rate and no curative treatment options available (Andtbacka et al. 2015; Hertzman Johansson and Egyhazi 2014; Hinrichs and Rosenberg 2014; Postow et al. 2015). We have extensively studied analogues of α-melanocyte-stimulating hormone (αMSH) for MC1R-targeted imaging and therapy. We have developed ^68^Ga or ^18^F labeled cyclic αMSH (CCZ01048, CCZ01064, CCZ01096) for PET imaging in preclinical models of mouse or human melanoma (Zhang et al. 2020; Zhang et al. 2019; Zhang et al. 2018; Zhang et al. 2017). In all cases, tracers achieved good tumor visualization in PET images with excellent tumor-to-normal tissue ratios. Moreover, we recently evaluated ^225^Ac labeled αMSH derivatives, which also exhibited excellent tumor-to-normal tissue ratios (Ramogida et al. 2019; Yang et al. 2020). Knowing the potential of tumor accumulation and internalization, and low uptake in normal tissues, we were interested in labeling an αMSH peptide with ^64^Cu to assess the unique theranostic power of ^64^Cu. [^64^Cu]Cu-αMSH peptide will allow for real-time monitoring of physiological processes during therapy studies, and when coupled with the longer half-life of ^64^Cu over other PET isotopes (i.e. ^68^Ga and ^18^F), it will enable later time points for imaging and biodistribution studies, and a more accurate assessment of the longer-term biological fate of both peptide and radionuclide.

## Results and discussions

The chelator synthesis started with the cyclen-xPy (x = 1–4, compounds **1**–**4**, Scheme 1). All derivatives were synthesized using a single reaction followed by purification by HPLC. Each compound was then reacted with tert-butyl bromoacetate and deprotected to yield the final product DOTA-xPy (x = 1–3, compounds **5**–**7**) (Subat and König 2001; Veiga et al. 2013). Compounds cyclen-1Py (Aime et al. 1999; Aoki et al. 2004; Subat et al. 2007), cyclen-2Py (El Hajj et al. 2009), cyclen-4Py (Natrajan et al. 2010) and DOTA-1Py (Aime et al. 1999) were previously reported, while compounds cyclen-3Py, DOTA-2Py and DOTA-3Py are new compounds to the best of our knowledge. DOTA-1Py and DOTA-3Py were obtained as a single compound. DOTA-2Py could potentially maintain both cis- and trans- stereoisomer configurations. Because ^1^H NMR of cyclen-2Py indicated a cis- derivative, DOTA-2Py was assumed to be cis-. The complexation of ^nat^Cu to DOTA-xPy (x = 1–3) was confirmed by IR and UV-vis spectra (Fig. S1 and S2 in Supporting Information). In the UV-vis spectra, we observed increased absorbance between 200 and 400 nm, with a new shoulder peak emerging at around 290 nm, suggesting metal complexation formation. A new peak at 720–740 nm and the blue color of the product indicated the formation of Cu(II). Also, for all three chelates, UV-vis spectra showed saturation at 1.2 eq. Cu(II), suggesting the complex has 1:1 Cu(II):chelate ratio. Infrared (IR) analysis showed the decrease of the stretching absorption at 1700 cm^− 1^ for C=O and 1200 cm^− 1^ for C-N (Spreckelmeyer et al. 2017), indicating that both COO and pyridyl moieties bind to the metal. In attempting to interpret the coordination geometry, we considered both the relative frequency and intensities of both asymmetric (ν_as_, ~ 1600 cm^− 1^) and symmetric (ν_s_, ~ 1400 cm^− 1^) COO stretching vibrations for the free and bound chelates (Berestova et al. 2017). For the DOTA-1Py and -2Py chelates, we observed the evolution of dual maxima upon metal binding, suggesting there exist multiple binding configurations for the COO moieties around the metal. That said, the spectroscopic data did not allow for the elucidation of a definitive orientation of binding motifs around the metal. Future studies will focus on a full structural analysis to aid in determining the coordination behavior for these chelates. The labeled peptide [^64^Cu]Cu-DOTA-2Py-αMSH showed a double peak on HPLC gamma trace under isocratic conditions, which could indicate the formation of stereoisomers upon metal coordination.

**Fig. 2:**
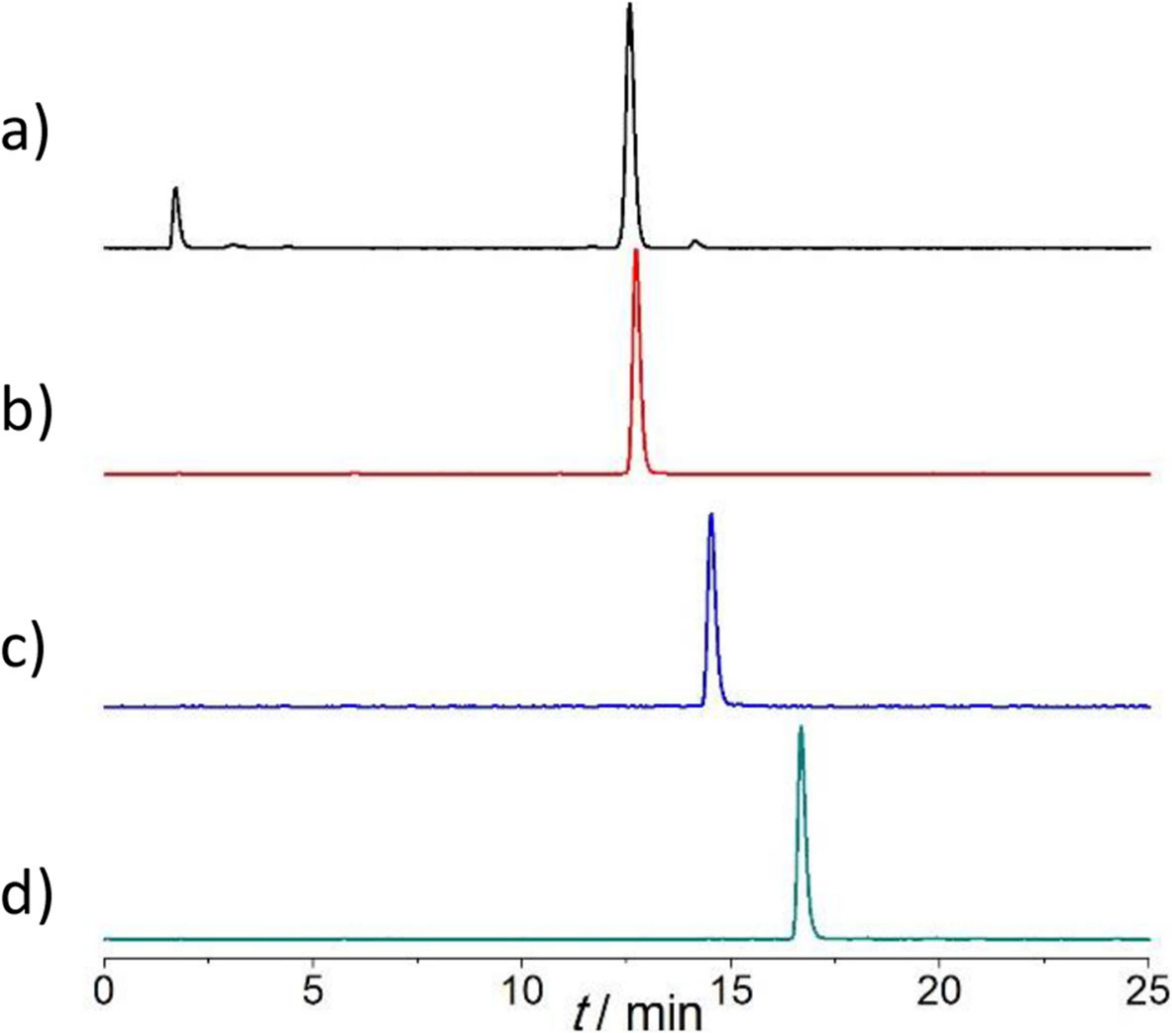
Radio-HPLC chromatogram of ^64^Cu labeling of (**a**) DOTA-1Py (retention time, t_R_ = 12.5 min), (**b**) DOTA-2Py (t_R_ = 12.7 min), (**c**) DOTA-3Py (t_R_ = 14.5 min), and (**d**) cyclen-4Py (t_R_ = 16.7 min) at ligand concentration 1 × 10^− 3^ M (100 nmol ligand, 3 MBq ^64^Cu) and ambient temperature

**Fig. 3:**
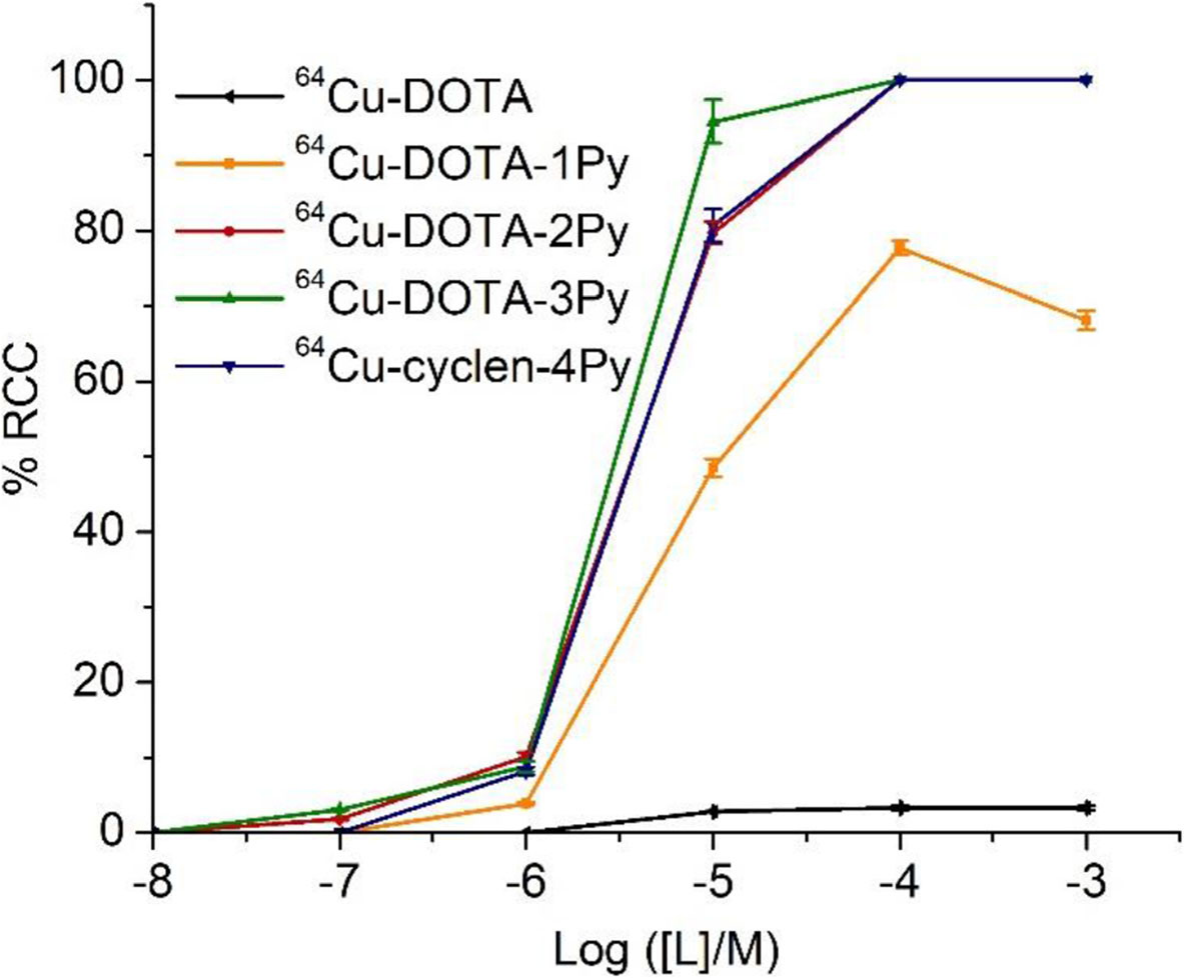
^64^Cu labeling conversion at various ligand concentrations for DOTA, DOTA-1Py, DOTA-2Py, DOTA-3Py, and cyclen-4Py (*n* = 3) at ambient temperature and 5 min reaction time

^64^Cu was produced by proton irradiation of enriched ^64^Ni solid metal targets using TRIUMF’s TR13 cyclotron. After acid dissolution and purification by cation exchange resin (AG1-X8), ^64^Cu was obtained in dilute HCl. ^64^Cu labeling of DOTA, DOTA-1Py, DOTA-2Py, DOTA-3Py, and cylen-4Py were examined at various ligand concentrations. The results (Fig. 2 and Fig. 3) indicated that the subsequent addition of pyridine moiety served to enhance ^64^Cu labeling conversion in general. While DOTA showed little complexation with ^64^Cu at ambient temperature even at ligand concentrations of 1 × 10^− 3^ M (100 nmol ligand, 3 MBq ^64^Cu), having one, two or three pyridyls significantly improved radiochemical conversion (RCC), with DOTA-3Py demonstrating efficient complexation of ^64^Cu (RCC close to 95%) at ligand concentrations of 1 × 10^− 5^ M (1 nmol ligand, 3 MBq ^64^Cu) and at ambient temperature. The differences in ^64^Cu labeling performance between DOTA-2Py and -4Py were not significant.

**Fig. 4:**
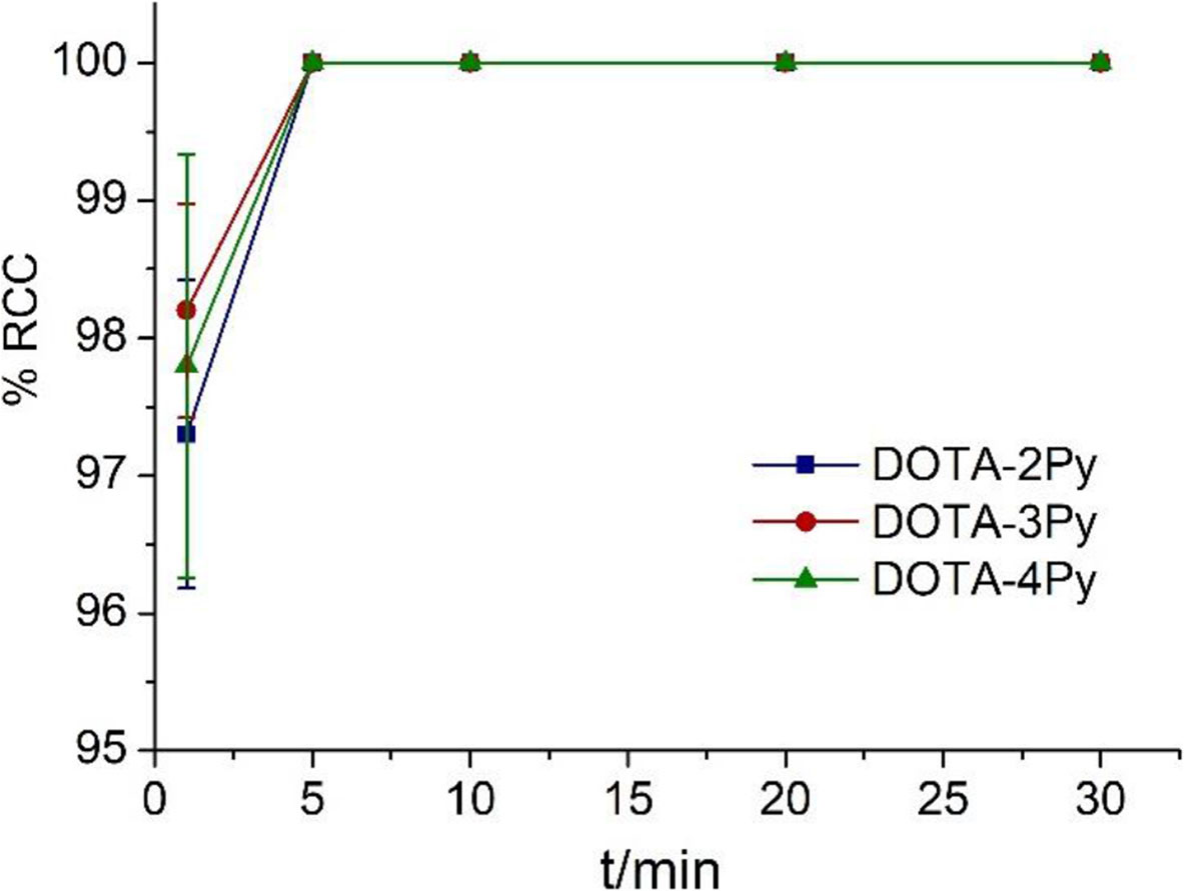
^64^Cu labeling conversion of DOTA-2Py, DOTA-3Py and cyclen-4Py at 1, 5, 10, 20 and 30 min performed with ligand concentration of 1 × 10^− 4^ M (10 nmol ligand, 3 MBq ^64^Cu) and ambient temperature (*n* = 3)

We then studied the reaction kinetics for labeling DOTA-2Py, −3Py and -4Py at 1 × 10^− 4^ M (10 nmol ligand, 3 MBq ^64^Cu, DOTA-1Py not studied because the labeling was less efficient). Under the same conditions as described above, the reactions were monitored at 1, 5, 10, 20 and 30 min by radioTLC. All three chelators demonstrated near quantitative metal incorporation within 5 min at ambient temperature (Fig. 4).

The stability of [^64^Cu]Cu-DOTA-2Py, −3Py and -4Py were examined in phosphate buffer and human serum at 1 h and 24 h at 37 °C. All three ligands were largely stable (> 94%) in both phosphate buffer and human serum in 24 h (Table 1).

**Table 1:** Stability of [^64^Cu]Cu-DOTA-xPy (x = 2–4) and [^64^Cu]Cu-DOTA-xPy-MSH (x = 1–3) in phosphate buffer and human serum at 37 °C

Radio-ligand	Phosphate buffer/%		Human serum/%	
	1 h	24 h	1 h	24 h
[^64^Cu]Cu-DOTA-2Py	99.2 ± 0.1	99.1 ± 0.1	98.7 ± 0.3	94.6 ± 0.1
[^64^Cu]Cu-DOTA-3Py	99.4 ± 0.3	99.2 ± 0.2	99.0 ± 0.1	96.1 ± 0.2
[^64^Cu]Cu-cyclen-4Py	99.1 ± 0.1	99.0 ± 0.2	98.5 ± 0.1	97.2 ± 0.3
[^64^Cu]Cu-DOTA-1Py-αMSH	99.3 ± 0.1	98.2 ± 0.2	98.4 ± 0.2	96.5 ± 0.3
[^64^Cu]Cu -DOTA-2Py-αMSH	99.1 ± 0.04	98.6 ± 0.3	99.4 ± 0.2	98.3 ± 0.2
[^64^Cu]Cu -DOTA-3Py-αMSH	99.2 ± 0.2	99.3 ± 0.2	98.1 ± 0.1	97.7 ± 0.2

NOTA has been the widely used chelate for labeling with ^64^Cu as it forms kinetically stable complexes (Cooper et al. 2012). For comparison, we examined the kinetic inertness of [^64^Cu]Cu-DOTA-2Py, −3Py and -4Py using NOTA as the transchelation challenge reagent. After the formation of [^64^Cu]Cu-DOTA-2Py, −3Py and -4Py at ligand concentration of 1 × 10^− 4^ M (10 nmol ligand, 3 MBq ^64^Cu), 1000 eq. of *p-*SCN-Bn-NOTA were added and the mixture was monitored by radio-HPLC over 4 h. During this time, no transchelation was observed for all three ^64^Cu complexes, indicating the kinetic inertness of [^64^Cu]Cu-DOTA-2Py, −3Py and -4Py. As control experiments, *p-*SCN-Bn-NOTA was labeled with ^64^Cu, and 1000 eq. of DOTA-2Py, −3Py or -4Py were added and no transchelation was observed for up to 4 h. Interestingly, when ^64^Cu was added to an equimolar mixture (1 × 10^− 4^ M ligand) of DOTA-xPy (x = 2–4) and *p-*SCN-Bn-NOTA, [^64^Cu]Cu-DOTA-2Py, −3Py and -4Py were formed with RCYs of 20%, 19% and 28%, while [^64^Cu]Cu-*p-*SCN-Bn-NOTA formed at RCYs of 78%, 79% and 70%, respectively, indicating DOTA-xPy (2–4) chelators may have slower kinetics compared to NOTA.

**Fig. 5:**
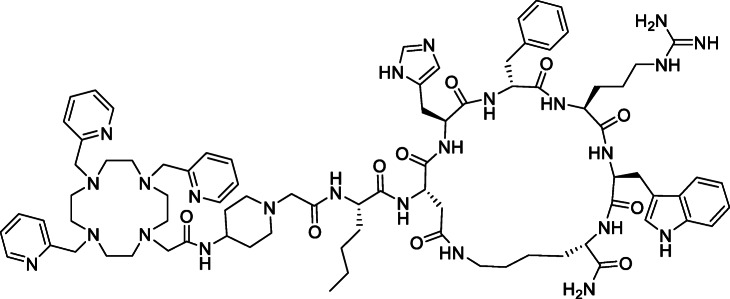
Chemical structure of DOTA-3Py-αMSH

With radiolabeling efficiency, stability and kinetic inertness of the Cu-complexes established, we attached DOTA-1Py, −2Py, −3Py to an αMSH peptide through a carboxylic moiety of each chelator (Fig. 5). Although DOTA-1Py-αMSH was expected to complex ^64^Cu less efficiently, it was included to study the trend with increasing pyridyls. The peptide was synthesized via the step-wise addition of amino acids followed by on-resin cyclization. The final Fmoc protecting group was removed by mixing with HOBt, HBTU and isopropyl ethylamine and agitated at ambient temperature overnight. The deprotection and purification by HPLC was done by standard procedures (Zhang et al. 2017) and characterized by mass spectrometry.

**Fig. 6:**
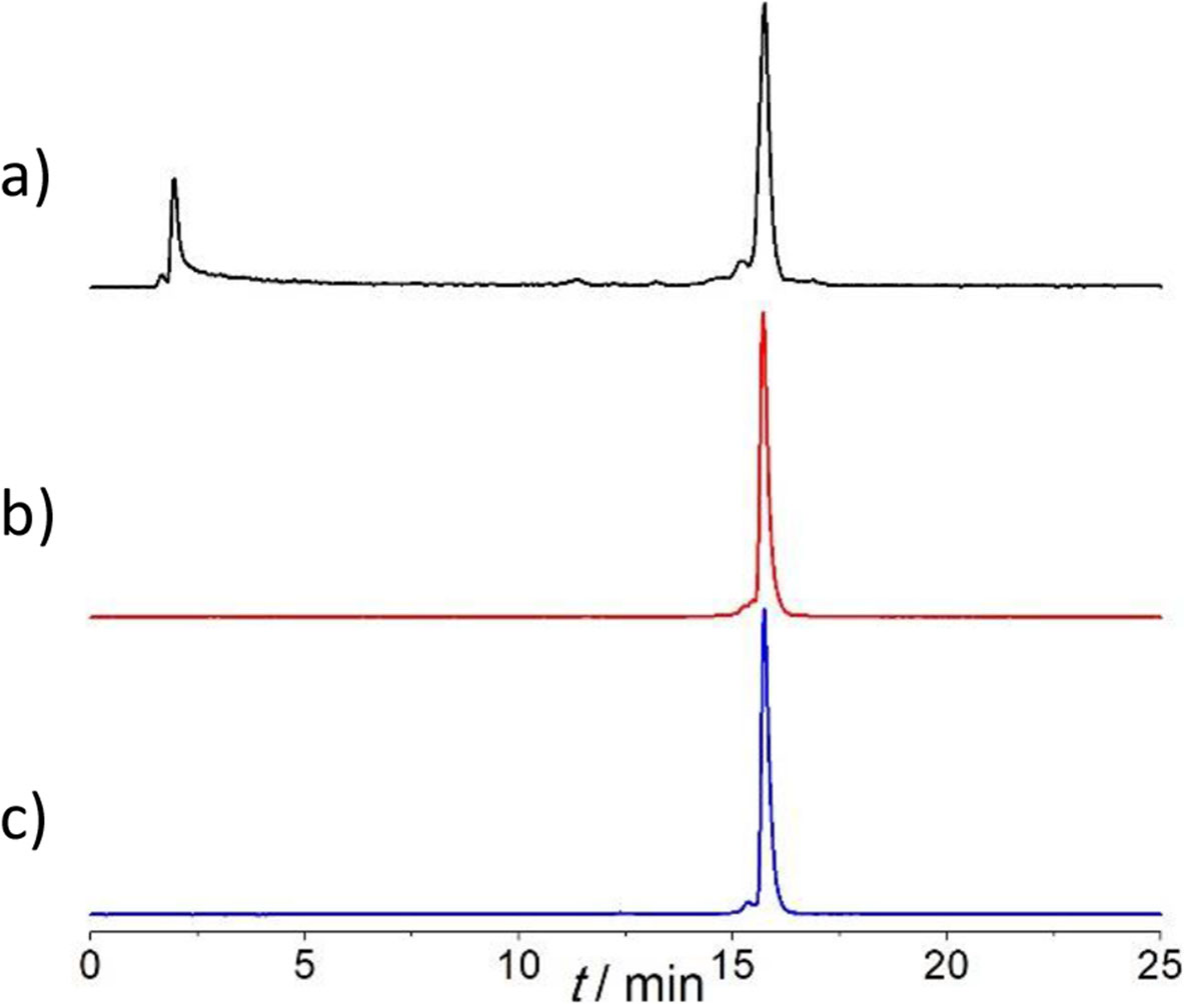
Radio-HPLC chromatograms of (**a**) [^64^Cu]Cu-DOTA-1Py-αMSH (RCY = 66%), (**b**) [^64^Cu]Cu-DOTA-2Py-αMSH, and (**c**) [^64^Cu]Cu-DOTA-3Py-αMSH

Radiolabeling was performed under similar conditions as discussed for the chelators themselves. [^64^Cu]CuCl_2_ was mixed with DOTA-xPy-αMSH in MES buffer (0.4 M, pH 6.2) and allowed to sit at ambient temperature. At 1 × 10^− 5^ M peptide concentration (1 nmol), labeling with 3 MBq of ^64^Cu was quantitative after 15 min for DOTA-2Py-αMSH and DOTA-3Py-αMSH and 66% for DOTA-1Py-αMSH (Fig. 6). The resulting [^64^Cu]Cu-DOTA-xPy-αMSH (x = 1–3) complexes were stable in both phosphate buffer and human serum at 37 °C for 24 h (radiochemical purity, RCP > 96%, Table 1).

Modifications of peptides may adversely impact target binding affinity (Di 2015). We studied the binding affinity of ^nat^Cu-DOTA-xPy-αMSH (x = 1–3) and found that binding of all three peptides towards MC1R remained in the in low nanomolar level (Table 2), although lower than Ga-DOTA-αMSH complex (K_i_ = 0.31 ± 0.06 for CCZ01048) (Zhang et al. 2017).

**Table 2:** Inhibition constants (K_i_) of ^nat^Cu-DOTA-xPy-αMSH (x = 1–3) towards MC1R (*n* = 3)

	^nat^Cu-DOTA-1Py-αMSH	^nat^Cu-DOTA-2Py-αMSH	^nat^Cu-DOTA-3Py-αMSH
K_i_ (nM)	2.34 ± 0.91	2.11 ± 0.55	3.72 ± 1.38

## Conclusion

We have synthesized a new set of chelators DOTA-1Py, −2Py, −3Py and -4Py and demonstrated that substitution of the carboxylic groups with one or two pyridine groups on a DOTA backbone can significantly improve the binding affinity towards ^64^Cu. We covalently attached the chelators to an αMSH peptide and demonstrated that DOTA-xPy-αMSH (x = 1–3) can be quantitatively labeled by ^64^Cu at ambient temperature. Moreover, all three ^nat^Cu-DOTA-xPy-αMSH (x = 1–3) retained good binding affinity towards MC1R. Metabolic stability and biodistribution evaluation of [^64^Cu]Cu-DOTA-xPy-αMSH (x = 1–3) is underway.

## Material and methods

### General

All reactions were carried out with commercial solvents and reagents that were used as received. Concentration and removal of trace solvents was done via a Büchi rotary evaporator using dry ice/acetone condenser, and vacuum applied from an aspirator or Büchi V-500 pump. Nuclear magnetic resonance (NMR) spectra were recorded using D_2_O or DMSO-d_6_ or MeOD-d_4_. Signal positions are given in parts per million from tetramethylsilane (δ = 0) and were measured relative to the signal of the solvent. ^1^H NMR spectral data are tabulated in the order: multiplicity (s, singlet; d, doublet; t, triplet; q, quartet; quint, quintet; m, multiplet). NMR spectra were recorded on a Bruker 400 (400 MHz) or a Bruker 300 (300 MHz) spectrometer at 25 °C unless otherwise noted. MS spectra was recorded on a Waters ZQ equipped with ESCI ion source (low resolution), a Kratos Concept IIHQ (high resolution) or a Bruker Autoflex MALDI-TOF. High performance liquid chromatography (HPLC) of radiolabeled samples was performed on an Agilent 1200 series instrument equipped with a diode array detector and Raytest GABI Star scintillation detector. Radioactivity was measured on a Capintec 55TR dose calibrator.

### Chelator synthesis

Compounds cyclen-1Py (**1**) (Aime et al. 1999; Aoki et al. 2004; Subat et al. 2007), cyclen-2Py (**2**) (El Hajj et al. 2009), cyclen-4Py (**4**) (Natrajan et al. 2010) and DOTA-1Py (**5**) (Aime et al. 1999) are previously reported and we synthesized with a modified procedure. Compound **2′** was co-produced with **2**.

Synthesis of cyclen-1Py (**1)**, cyclen-2Py (**2, 2′**), cyclen-3Py (**3**), and cyclen-4Py (**4**): To a suspension of cyclen (172.3 mg, 1 mmol) and K_2_CO_3_ (552 mg, 4 mmol) in anhydrous acetonitrile (25 mL), a solution of 2-(bromomethyl)pyridine hydrobromide (329 mg, 1.3 mmol) in 10 mL anhydrous acetonitrile was added dropwise over a period of 1 h at 0 °C and the reaction mixture was allowed to stand at room temperature overnight. After the reaction was complete, the precipitate was filtered off and the solvent was removed by evaporation. The crude products were purified on reverse phase semi-preparative HPLC using Phenomenex Luna C18 (250 mm × 100 mm) column at 3 mL/min with the following method: A: H_2_O with 0.1% TFA, B: CH_3_CN with 0.1% TFA; 0–20 min, 100% A isocratic; 20-30 min, 100%–85% A, 0%–15% B; 30-45 min, 85% A, 15% B. The fraction at 6.3 min, 15.5 min and 33.0 min were collected and lyophilized to yield **1** as a colorless solid (40 mg, 15%), **2** (**2′**) as a yellowish solid (63 mg, 18%) and **3** as a yellowish solid (76 mg, 17%), respectively.

1-(Pyridin-2-ylmethyl)-1,4,7,10-tetraazacyclododecane (cyclen-1Py, **1**): ^1^H NMR (300 MHz, D_2_O) δ 8.75 (dd, *J* = 5.9, 1.6 Hz, 1H), 8.55 (td, *J* = 8.0, 1.6 Hz, 1H), 8.09–7.93 (m, 2H), 4.16 (s, 2H), 3.27 (t, *J* = 5.2 Hz, 4H), 3.20–3.10 (m, 4H), 3.01–2.87 (m, 8H). ^13^C NMR (126 MHz, MeOD) δ 158.56, 149.53, 140.87, 125.18, 125.09, 58.38, 51.06, 46.07, 44.19, 44.01. HR-MS: calcd. For C_24_H_26_N_5_ ([M + H]^+^): 264.2188; found: 264.2112.

1,4-Di(pyridin-2-ylmethyl)-1,4,7,10-tetraazacyclododecane and 1,2-Di(pyridin-2-ylmethyl)-1,4,7,10-tetraazacyclododecane (cyclen-2Py, **2** and **2′**): ^1^H NMR (300 MHz, D_2_O) δ 8.65–8.55 (m, 2H), 8.30 (td, *J* = 7.9, 1.7 Hz, 2H), 7.88–7.71 (m, 4H), 4.08 (s, 4H), 3.43 (s, 4H), 3.32 (dd, *J* = 6.7, 4.3 Hz, 4H), 3.08–2.92 (m, 8H). ^13^C NMR (126 MHz, MeOD) δ 161.92 (q, *J* = 36.5 Hz, CF_3_*C*O_2_H) 154.44, 148.71, 141.43, 126.23, 125.61, 117.59 (q, *J* = 291.1 Hz, *C*F_3_CO_2_H) 57.24, 51.90, 51.51, 44.51, 43.80. ESI-MS calcd. For C_20_H_31_N_6_ ([M + H]^+^): 355.3; found: 355.3.

1,4,7-Tri(pyridin-2-ylmethyl)-1,4,7,10-tetraazacyclododecane (cyclen-3Py, **3**): ^1^H NMR (500 MHz, MeOD) δ 8.63 (dd, J = 5.4, 1.6 Hz, 2H), 7.93 (td, J = 7.8, 1.7 Hz, 2H), 7.81 (td, J = 7.8, 1.8 Hz, 1H), 7.62 (d, J = 7.9 Hz, 2H), 7.57 (dd, J = 5.1, 1.6 Hz, 1 H), 7.52–7.45 (m, 2H), 7.39 (d, J = 7.8 Hz, 1H), 7.23 (dd, J = 7.5, 4.9 Hz, 1H), 4.54 (s, 2H), 4.06 (s, 4H), 3.63 (t, J = 5.3 Hz, 4H), 3.42 (t, J = 5.1 Hz, 4H), 3.23 (t, J = 5.3 Hz, 4H), 3.09–2.96 (m, 4H). ^13^C NMR (126 MHz, MeOD) δ 162.02 (CF_3_*C*O_2_H), 161.73 (CF_3_*C*O_2_H), 161.44, 156.25, 150.49, 149.82, 148.33, 141.39, 139.42, 126.33, 125.64, 125.14, 118.65 (*C*F_3_CO_2_H), 116.35 (*C*F_3_CO_2_H), 58.18, 57.11, 52.65, 50.12, 43.73. HR-MS: calcd. For C_26_H_36_N_7_ ([M + H]^+^): 446.3032; found: 446.2989.

Preparation of **cyclen-4Py**: A mixture of cyclen (35 mg, 0.2 mmol) and K_2_CO_3_ (414 mg, 3 mmol) in dry acetonitrile (15 mL) was placed in an ice bath and then a solution of 2-(bromomethyl)pyridine (190 mg, 0.75 mmol) in 10 mL dry CH_3_CN was added dropwise over a period of 1 h. The resulting mixture was stirred for additional 10 h. The precipitate was filtered, and the filtrate concentrated in vacuo to give the crude product which was further purified by HPLC using the same method described above. ^1^H NMR (300 MHz, D_2_O) δ 8.57 (d, *J* = 5.3 Hz, 4H), 8.00 (td, *J* = 7.8 Hz, 1.1 Hz, 4H), 7.80 (d, *J* = 7.5 Hz, 4H), 7.66 (t, *J* = 6.3 Hz, 4H), 4.36 (s, 8H), 3.45 (s, 16H). ESI-MS: calcd. For C_32_H_41_N_8_ ([M + H]^+^): 537.3; found: 537.3.

Preparation of **DOTA-1Py**: A solution of tert-butyl bromoacetate (59 mg, 0.3 mmol) in dry CH_3_CN (5 mL) was added dropwise to a mixture of cyclen-1py (26 mg, 0.1 mmol), K_2_CO3 (83 mg, 0.6 mmol) and KI (10 mg, 0.06 mmol) in dry CH_3_CN (10 mL). The resulting mixture was stirred at room temperature for 1 h and then refluxed overnight. After cooling to room temperature, the mixture was filtered, and the residue washed with dry CH_3_CN. The filtrate was collected and evaporated to yield the title compound as a white solid. The solid was dissolved in 10 mL of mixed solvent containing TFA (9.5 mL), TIPS (0.25 mL) and H_2_O (0.25 mL). The reaction mixture was stirred at room temperature for 2 h. The solvent was removed by air flow and the residue was dissolved in CH_3_CN/H_2_O (1/1). The crude product was purified on reverse phase semi-preparative HPLC using Phenomenex Luna C18 (250 mm × 100 mm) column at 3 mL/min with the following method: A: H_2_O with 0.1% TFA, B: CH_3_CN with 0.1% TFA; 0-5 min, 100% A isocratic; 5–25 min, 100%–60% A, 0%–40% B. The fraction at 6.7 min was collected and lyophilized to give the title compound as a colorless oil (32 mg, 74%). ^1^H NMR (600 MHz, D_2_O) δ 8.69 (ddd, *J* = 5.9, 1.6, 0.6 Hz, 1H), 8.46 (td, *J* = 7.9, 1.6 Hz, 1H), 8.03 (d, *J* = 8.0 Hz, 1H), 7.94 (ddd, *J* = 7.3, 5.9, 1.3 Hz, 1H), 4.09 (s, 2H), 3.89 (d, *J* = 15.8 Hz, 2H), 3.68 (d, *J* = 16.4 Hz, 4H), 3.47–3.44 (m, 4H), 3.35 (s, 2H), 3.31–3.28 (m, 2H), 3.15 (s, 2H), 3.06–3.02 (m, 2H), 2.92–2.86 (m, 4H). ^13^C NMR (151 MHz, D_2_O) δ 175.48, 169.03, 162.83 (q, *J* = 35.6 Hz, CF_3_*C*O_2_H), 150.13, 147.53, 143.16, 129.61, 128.39, 126.93, 116.26 (q, *J* = 291.7 Hz, *C*F_3_CO_2_H), 55.34, 53.60, 52.87, 52.05, 50.36, 48.15, 48.01. ESI-MS: calcd. For C_20_H_32_N_5_O_6_ ([M + H^+^]): 438.2; found: 438.2.

Preparation of **DOTA-2Py**: cyclen-2py (39 mg, 0.2 mmol) was dissolved in dry acetonitrile (10 mL), K_2_CO_3_ (55 mg, 0.4 mmol) and KI (7 mg, 0.04 mmol) was added to the solution. Then a solution of tert-butyl bromoacetate (35 mg, 0.1 mmol) in dry CH_3_CN (5 mL) was added dropwise. The mixture was stirred at room temperature for 1 h and then refluxed overnight. After cooling to room temperature, the mixture was filtered, and the filtrate was evaporated to yield the title compound as a yellowish solid. The solid was dissolved in 10 mL of mixed solvent containing TFA (9.5 mL), TIPS (0.25 mL) and H_2_O (0.25 mL). The reaction mixture was stirred at room temperature for 2 h. The solvent was removed by air flow and the residue was dissolved in CH_3_CN/H_2_O (1/1) for further HPLC purification using the same method described above. The fraction from 14 to 14.5 min was collected and lyophilized to give the title compound as colorless oil (36 mg, 77%). ^1^H NMR (500 MHz, MeOD) δ 8.44 (dd, J = 5.3, 1.7 Hz, 2H), 7.79 (td, J = 7.7, 1.7 Hz, 2H), 7.55 (s, 2H), 7.35–7.31 (m, 2H), 4.36–4.08 (m, 4H), 3.73 (s, 4H), 3.53–2.95 (m, 18H, including MeOD). ^13^C NMR (75 MHz, DMSO) δ 170.77, 158.86 (CF_3_*C*OOH), 158.43 (CF_3_*C*OOH), 153.45, 149.07, 137.97, 124.36, 123.76, 119.20 (*C*F_3_COOH), 115.25 (*C*F_3_COOH), 57.12, 53.93, 50.00, 49.75, 49.44. HR-MS: calcd. For C_24_H_35_N_6_O_4_ ([M + H]^+^): 471.2720; found: 471.2558.

Preparation of **DOTA-3Py**: To a suspension of cyclen-3py (45 mg, 0.1 mmol), K_2_CO_3_ (28 mg, 0.2 mmol) and KI (3 mg, 0.02 mmol) in dry acetonitrile (15 mL), a solution of tert-butyl bromoacetate (20 mg, 0.1 mmol) in dry CH_3_CN (5 mL) was added. The resulting mixture was refluxed overnight. The precipitated solids were removed by filtration and the filtrate was concentrated to give the title compound as a yellowish solid. The solid was dissolved in 10 mL of mixed solvent containing TFA (9.5 mL), TIPS (0.25 mL) and H_2_O (0.25 mL). The reaction mixture was stirred at room temperature for 2 h. The solvent was removed by air flow and the residue was dissolved in CH_3_CN/H_2_O (1/1) for further HPLC purification using the same method described above. The fraction at 15.2 min was collected and lyophilized to give the title compound as colorless oil (41 mg, 81%). ^1^H NMR (600 MHz, D_2_O) δ 8.60 (d, *J* = 5.7 Hz, 2H), 8.50 (d, *J* = 4.9 Hz, 1H), 8.37–8.30 (m, 2H), 8.02 (s, 2H), 7.83 (td, *J* = 7.8, 1.6 Hz, 1H), 7.73 (t, *J* = 7.1 Hz, 2H), 7.45–7.39 (m, 1H), 7.32 (s, 1H), 4.32 (s, 2H), 4.16–3.91 (m, 4H), 3.83 (s, 2H), 3.73–3.42 (m, 8H), 3.30–2.94 (m, 8H). ^13^C NMR (151 MHz, D_2_O) δ 178.19, 162.97 (q, *J* = 34.7 Hz, CF_3_*C*OOH), 156.69, 156.64, 147.95, 147.90, 147.82, 147.74, 147.64, 138.80, 138.78, 138.57, 124.37, 124.23, 124.20, 123.94, 123.65, 123.41, 116,3 (q, *J* = 276.3 Hz, *C*F_3_COOH) 59.88, 59.68, 59.15, 58.40, 53.02, 52.09, 51.86, 51.46, 51.25, 51.00, 49.18. HR-MS: calcd. For C_28_H_38_N_7_O_2_ ([M + H]^+^): 504.3087; found: 504.2943.

### IR and UV-vis titration of DOTA-xPy (x = 1–3)

A PerkinElmer Fourier transfer-infrared spectrometer (FT-IR) was used to obtain the spectra of three chelators and their respective metal complexes with Cu^2+^ metal ions, at 298 K, covering wavenumbers 650–4000 cm^− 1^. The six samples had been dried to be solid samples as TFA salts. Samples were re-dissolved in ACN and dried three times to remove extra TFA. Approximately 2–5 mg of each sample was used to cover the 2 mm diameter crystal on the attenuated total reflectance (ATR) top plate. UV-vis absorbance was recorded on an Agilent Cary100 UV-visible spectrophotometer. 5 mg of each chelator was dissolved in water to make a solution of 0.26–29 mM. 0.2 eq. of Cu(II) aqueous solution (trace metal basis) was added for each titration. UV-vis spectra at 200–800 nm was recorded using a 1 cm pathlength quartz cell.

### Production of ^64^Cu

Isotopically enriched ^64^Ni metal powder (50 mg) was dissolved in HCl. Aqueous ammonia was added to adjust the pH to ~ 9. ^64^Ni is then electroplated onto a rhodium backing disk (35 mm diameter, 1 mm thickness) using a small custom plating apparatus overnight. The plated nickel layer was approximately 8 mm diameter × 110 μm thickness. The target was irradiated for 1 h at 10 μA proton current 13 MeV and left in the cyclotron for 2 h to allow for the decay of the short-lived isotopes. The major co-produced isotopes were ^61^Co (2.9%) and ^103^Pb (2.0%) after cool-down. The ^64^Ni layer of the target was dissolved in hot concentrated HCl. After evaporation to dryness, the residue was re-dissolved in 6 M HCl and loaded onto a 1 cm AG1-X8 solid extraction column pre-conditioned with 6 M HCl. The column was washed with 6 M HCl (10 mL) and ^64^Cu was eluted with milli-Q water. Radionuclide purity (> 99%) was confirmed by gamma spectroscopy.

### RadioTLC

RadioTLC was carried out using BioScan system 200 Image Scanner. iTLC-SA plates used as stationary phase, developed using 0.2 M EDTA (pH 5). Under these conditions uncomplexed ^64^Cu^II^ migrates with the solvent front (R_f_ = 1), while the ^64^Cu-complex remains at the baseline (R_f_ = 0).

### Labeling of DOTA, DOTA-1Py, DOTA-2Py, DOTA-3Py and cyclen-4py (cyclen-4Py) with ^64^Cu

To a solution of ligand (10 μL, 1 × 10^− 3^ M) containing MES buffer (83 μL, 0.4 M, pH = 6.2) in an Eppendorf tube was added [^64^Cu]CuCl_2_ (7 μL 3 MBq). Final concentration of the ligand was 1 × 10^− 4^ M. The resulting mixture was kept at room temperature for 30 min. After the reaction, radiochemical purity of the radio-ligands was confirmed by both radio-HPLC and radio-iTLC. HPLC method: Phenomenex Luna C18 column (150 mm × 4.6 mm), 1 mL/min. A: H_2_O with 0.1% TFA, B: CH_3_CN with 0.1% TFA, 0–5 min: 100% A isocratic; 5–25 min: 100%–60% A, 0–40% B. [^64^Cu]Cu-DOTA-1Py, *t*_R_ = 12.5 min; [^64^Cu]Cu-DOTA-2Py, *t*_R_ = 12.7 min; [^64^Cu]Cu-DOTA-3Py, *t*_R_ = 14.5 min; [^64^Cu]Cu-cyclen-4Py, *t*_R_ = 16.7 min.

### Concentration dependence ^64^Cu labeling

To aqueous solution of ligands (10 μL, 1 × 10^− 2^ M, 1 × 10^− 3^ M, 1 × 10^− 4^ M, 1 × 10^− 5^ M, 1 × 10^− 6^ M, 1 × 10^− 7^ M) in MES buffer (83 μL, 0.4 M, pH = 6.2) was added [^64^Cu]CuCl_2_ (3 MBq, 7 μL of 0.01 M HCl). Final concentrations of the ligand were 1 × 10^− 3^ M, 1 × 10^− 4^ M, 1 × 10^− 5^ M, 1 × 10^− 6^ M, 1 × 10^− 7^ M, 1 × 10^− 8^ M. The mixtures were kept at room temperature for 15 min and then analyzed by radio-HPLC and radio-iTLC using the conditions described above.

### Kinetics of radiolabeling studies

To an aqueous solution of ligand (10 μL, 1 × 10^− 3^ M) in MES buffer (83 μL, 0.4 M, pH = 6.2) was added [^64^Cu]CuCl_2_ (3 MBq, 7 μL in 0.01 M HCl). The reaction mixture was kept at room temperature. Aliquots of the mixtures were analyzed by radio-HPLC and radio-iTLC using the conditions described above to determine the radiolabeling yields.

### Ligand competition radiolabeling

To equimolar aqueous mixtures of new ligand (10 μL, 1 × 10^− 3^ M DOTA-xPy (x = 1–4)) and commercially available chelator *p*-SCN-Bn-NOTA (10 μL, 1 × 10^− 3^ M NOTA-SCN) in MES buffer (73 μL, 0.4 M, pH = 6.2) was added [^64^Cu]CuCl_2_ (3 MBq, 7 μL in 0.01 M HCl). Final concentrations of new ligand and NOTA-SCN were 1 × 10^− 4^ M in the reaction mixtures. The mixtures were kept at room temperature for 15 min and then analyzed by radio-HPLC using the same method described above to determine the labeling ratio of radiolabeled new ligand and [^64^Cu]Cu-NOTA-SCN (*t*_R_ = 24.2 min).

### Ligand challenge studies

To a solution of ligand (10 μL, 1 × 10^− 3^ M) containing MES buffer (83 μL, 0.4 M, pH = 6.2) in an Eppendorf tube was added [^64^Cu]CuCl_2_ (7 μL 3 MBq). Final concentration of the ligand was 1 × 10^− 4^ M. After 10 min, full complexation of the ligand was checked by radio-HPLC and radio-iTLC. NOTA-SCN (1000-fold excess) dissolved in water (50 μL) was subsequently added to the reaction solution, and stirred for 4 h at ambient temperature. The mixture was assayed by radio-HPLC to determine the decomposition. As an opposing experiment, an aqueous solution of NOTA-SCN (10 μL, 1 × 10^− 3^ M) and [^64^Cu]CuCl_2_ (3 MBq, 7 μL in 0.01 M HCl) were subsequently added into MES buffer (83 μL, 0.4 M, pH = 6.2). After full complexation, DOTA-xPy (x = 1–4, 1000-fold excess) dissolved in water (50 μL) was added to the complex solution and the mixture was stirred for 4 h. The mixture was analyzed by radio-HPLC to determine the decomposition.

### In vitro stability of radio-ligands

Pre-formed ^64^Cu-labeled complexes (100 μL) were added to phosphate buffer (300 μL, pH 7.4) or human serum (300 μL). After 1 h or 24 h of incubation at 37 °C, the buffer samples were analyzed by semi-preparative HPLC. The serum samples were firstly treated with ethanol to precipitate and separate proteins (twice the volume of the mixture) and centrifuged for 1 min (13,200 rpm). The filtrate was decanted and further filtered (VWR sterile syringe filter, 0.2 μm PES), and subsequently analyzed by radio-iTLC using the conditions outlined above.

### Preparation of DOTA-1Py-αMSH, DOTA-2Py-αMSH and DOTA-3Py-αMSH

Peptide synthesis for Fmoc-Pip-Nle-CycMSH_hex_-resin (Fmoc-αMSH-resin) was performed as described in previously published procedures (Zhang et al. 2017). Subsequently, Fmoc was removed. (2-(1*H*-benzotriazol-1-yl)-1,1,3,3-tetramethyluronium hexafluorophosphate (HBTU, 5 eq.), hydroxybenzotriazole (HOBt, 5 eq.) and DOTA-xPy (x = 1–3, 5 eq.) were dissolved in DMF (minimum), then added to the resin (1 eq.) and initiated by the addition of N,N-diisopropylethylamine (15 eq.). Coupling was carried out for ~ 16 h at room temperature with shaking. The resin was washed extensively with DMF and DCM and solvent removed in a flow of N_2_. The peptide was cleaved and deprotected by soaking the resin with a mixture of TFA/TIPS/water/phenol (90/2.5/2.5/5) and shaking at room temperature for 3 h. After ether precipitation and filtration, the filtrate was collected and dried by N_2_. The residue was dissolved in CH_3_CN and water and purified by semi-preparative HPLC using Phenomenex Luna C18 (250 mm × 100 mm) column at 3 mL/min with the following method: A: H_2_O with 0.1% TFA, B: CH_3_CN with 0.1% TFA; 0–5 min, 90% A, 10% B; 5-25 min, 90%–10% A, 10%–90% B. DOTA-1Py-αMSH, *t*_R_ = 14.9 min; DOTA-2Py-αMSH *t*_R_ = 15.1 min; DOTA-3Py-αMSH *t*_R_ = 15.4 min.

MALDI-TOF MS: DOTA-1Py-αMSH: calcd. 1541.8 for [C_75_H_109_N_22_O_14_]^+^ ([M + H]^+^), found: 1542.0. DOTA-2Py-αMSH: calcd. 1574.9 for [C_79_H_112_N_23_O_12_]^+^ ([M + H]^+^), found: 1574.9. DOTA-1Py-αMSH: calcd. 1607.9 for [C_83_H_115_N_24_O_10_]^+^ ([M + H]^+^), found: 1607.9.

### Preparation of non-radioactive complexes (^nat^Cu-DOTA-Py-MSH, ^nat^Cu-DOTA-2Py-MSH and ^nat^Cu-DOTA-3Py-MSH)

A solution of peptide (200 μL, 1 × 10^− 3^ M) and CuSO_4_ (100 μL, 1 × 10^− 2^ M) in an Eppendorf tube was kept at room temperature for 2 h and subsequently purified by C-18 Sep-Pak (Waters). Non-radioactive title complexes were confirmed by ESI-MS: ^nat^Cu-DOTA-1Py-αMSH: calcd. 801.9 for [C_75_H_107_^63^CuN_22_O_14_]^2+^ ([M + 2H]^+^), found: 801.3. ^nat^Cu-DOTA-2Py-αMSH: calcd. 818.4 for [C_79_H_112_^63^CuN_23_O_12_]^+^ ([M + H]^2+^), found: 818.0. ^nat^Cu-DOTA-1Py-αMSH: calcd. 834.9 for [C_83_H_115_^63^CuN_24_O_10_]^+^ (M^2+^), found: 834.4. HPLC retention times were recorded using method: Phenomenex Luna C18 column (150 mm × 4.6 mm), 1 mL/min. A: H_2_O with 0.1% TFA, B: CH_3_CN with 0.1% TFA, 0–5 min: 100% A isocratic; 5–25 min: 100%–0% A, 0–100% B. ^nat^Cu-DOTA-1Py-αMSH, *t*_R_ = 15.6 min; ^nat^Cu-DOTA-2Py-αMSH, *t*_R_ = 16.3 min; ^nat^Cu-DOTA-3Py-αMSH, *t*_R_ = 15.4 min.

### ^64^Cu labeling of bioconjugates DOTA-1Py-αMSH, DOTA-2Py-COOH-αMSH and DOTA-3Py-αMSH

To a solution of bioconjugate (10 μL, 1 × 10^− 4^ M) in MES buffer (83 μL, 0.4 M, pH = 6.2) was added ^64^CuCl_2_ (3 MBq, 7 μL). Final concentration of the bioconjugate was 1 × 10^− 5^ M. The resulting mixture was kept at room temperature for 15 min. After the reaction, radiochemical purity of the radiolabeled bioconjugate was confirmed by both radio-HPLC and radio-iTLC. HPLC method: Phenomenex Luna C18 column (150 mm × 4.6 mm), 1 mL/min. A: H_2_O with 0.1% TFA, B: CH_3_CN with 0.1% TFA, 0–5 min: 100% A isocratic; 5–25 min: 100%–0% A, 0–100% B. [^64^Cu]Cu-DOTA-1Py-αMSH, *t*_R_ = 15.9 min; [^64^Cu]Cu-DOTA-2Py-αMSH, *t*_R_ = 16.7 min; [^64^Cu]Cu-DOTA-3Py-αMSH, *t*_R_ = 15.7 min.

### In vitro stability of radiolabeled peptides

Pre-formed ^64^Cu-labeled bioconjugate (100 μL) was mixed in phosphate buffer (300 μL, pH 7.4) or human serum (300 μL). After 1 h or 4 h of incubation at 37 °C, the buffer samples were analyzed by semi-preparative HPLC. The serum samples were firstly treated with ethanol to precipitate and separate proteins (twice the volume of the mixture) and centrifuged for 1 min (13,200 rpm). The filtrate was decanted and further filtered (VWR sterile syringe filter, 0.2 μm PES), and subsequently analyzed by radio-iTLC using the conditions outlined above.

### Binding affinity of ^nat^Cu-DOTA-xPy towards MC1R

K_i_ was measured using the same method previously reported (Zhang et al. 2017). Briefly, 5 × 10^5^ B16F10 cells/well were seeded in complete growth medium in a 24 well Poly-D-Lysine plate (Corning BioCoat™, Ref No. 354414) and grown to 85–90% confluency at standard conditions. The growth medium was replaced with reaction medium (RPMI, 2 mg/ml BSA, 20 mM HEPES) and the non-radioactive ligand added at a final concentration of 0.5 pM to 5 μM, along with 0.1 nM [^125^I]I-αMSH. The cells were incubated for 1 h at 25 °C with mild shaking. The supernatant was removed and cells were washed twice with ice-cold PBS. Cells were harvested with trypsin and counted on WIZARD 2480 gamma counter. The measurements were repeated in three separate experiments and reported as mean ± standard deviation.

## Supplementary Information

**Additional file 1.**

## Data Availability

The datasets used and/or analysed during the current study are available from the corresponding author on reasonable request.

## Abbreviations

ACN: Acetonitrile
αMSH: α-melanocyte-stimulating hormone
DOTA: 1,4,7,10-tetraazacyclododecane-1,4,7,10-tetraacetic acid
EDTA: Ethylenediamine tetraacetic acid
HBTU: (2-(1*H*-benzotriazol-1-yl)-1,1,3,3-tetramethyluronium hexafluorophosphate
HOBt: Hydroxybenzotriazole
HPLC: High performance liquid chromatography
iTLC: instant thin layer chromatography
MC1R: Melanocortin 1 receptor
MES: 2-(N-morpholino)ethanesulfonic acid
NOTA: 1,4,7-triazacyclononane-triacetic acid
TFA: Trifluoroacetic acid
TIPS: Triisopropyl silane

## References

[CR1] Aime S, Batsanov AS, Botta M, Howard JAK, Lowe MP, Parker D (1999). Structure and relaxivity of macrocyclic gadolinium complexes incorporating pyridyl and 4-morpholinopyridyl substituents. New J Chem.

[CR2] Anderson CJ, Ferdani R (2009). Copper-64 radiopharmaceuticals for PET imaging of cancer: advances in preclinical and clinical research. Cancer Biother Radiopharm.

[CR3] Andtbacka RHI, Kaufman HL, Collichio F, Amatruda T, Senzer N, Chesney J (2015). Talimogene laherparepvec improves durable response rate in patients with advanced melanoma. J Clin Oncol.

[CR4] Aoki S, Kagata D, Shiro M, Takeda K, Kimura E (2004). Metal chelation-controlled twisted intramolecular charge transfer and its application to fluorescent sensing of metal ions and anions. J Am Chem Soc.

[CR5] Berestova TV, Kuzina LG, Amineva NA, Faizrakhmanov IS, Massalimov IA (2017). Mustafin AG ATR-FTIR spectroscopic investigation of the cis- and trans-bis-(α-amino acids) copper(II) complexes. J Mol Struct.

[CR6] Cai Z, Anderson CJ (2014). Chelators for copper radionuclides in positron emission tomography radiopharmaceuticals. J Label Compd Radiopharm.

[CR7] Cooper MS, Ma MT, Sunassee K, Shaw KP, Williams JD, Paul RL (2012). Comparison of 64cu-complexing bifunctional chelators for radioimmunoconjugation: labeling efficiency, specific activity, and in vitro / in vivo stability. Bioconjug Chem.

[CR8] Czerwińska K, Machura B, Kula S, Krompiec S, Erfurt K, Roma-Rodrigues C (2017). Copper(II) complexes of functionalized 2,2′:6′,2′′-terpyridines and 2,6-di(thiazol-2-yl)pyridine: structure, spectroscopy, cytotoxicity and catalytic activity. Dalt Trans.

[CR9] De León-Rodríguez LM, Kovacs Z (2008). The synthesis and chelation chemistry of DOTA-peptide conjugates. Bioconjug Chem.

[CR10] Di Bartolo N, Sargeson AM, Smith SV (2006). New 64Cu PET imaging agents for personalised medicine and drug development using the hexa-aza cage. Org Biomol Chem.

[CR11] Di L (2015). Strategic approaches to optimizing peptide ADME properties. AAPS J.

[CR12] El Hajj F, Sebki G, Patinec V, Marchivie M, Triki S, Handel H (2009). Macrocycle-based spin-crossover materials. Inorg Chem.

[CR13] Gutfilen B, Souza SAL, Valentini G (2018). Copper-64: a real theranostic agent. Drug Des Dev Ther.

[CR14] Hertzman Johansson C, Egyhazi BS (2014). BRAF inhibitors in cancer therapy. Pharmacol Ther.

[CR15] Hinrichs CS, Rosenberg SA (2014). Exploiting the curative potential of adoptive T-cell therapy for cancer. Immunol Rev.

[CR16] Langbein T, Weber WA, Eiber M (2019). Future of theranostics: an outlook on precision oncology in nuclear medicine. J Nucl Med.

[CR17] Lee WS, Yeung CT, Sham KC, Wong WT, Kwong HL (2011). Chiral copper-bipyridine complexes: synthesis, characterization and mechanistic studies on asymmetric cyclopropanation. Polyhedron.

[CR18] Leussing DL, Hansen RC (1957). The copper(II)-pyridine complexes and their reaction with hydroxide ions. J Am Chem Soc.

[CR19] López MN, Pereda C, Ramírez M, Mendoza-Naranjo A, Serrano A, Ferreira A (2007). Melanocortin 1 receptor is expressed by uveal malignant melanoma and can be considered a new target for diagnosis and immunotherapy. Investig Ophthalmol Vis Sci.

[CR20] McCarthy DW, Shefer RE, Klinkowstien RE, Bass LA, Margeneau WH, Cutler CS (1997). Efficient production of high specific activity 64Cu using a biomedical cyclotron. Nucl Med Biol.

[CR21] Natrajan LS, Khoabane NM, Dadds BL, Muryn CA, Pritchard RG, Heath SL (2010). Probing the structure, conformation, and stereochemical exchange in a family of lanthanide complexes derived from tetrapyridyl-appended cyclen. Inorg Chem.

[CR22] Postow MA, Callahan MK, Wolchok JD (2015). Immune checkpoint blockade in Cancer therapy. J Clin Oncol.

[CR23] Ramogida CF, Robertson AKH, Jermilova U, Zhang C, Yang H, Kunz P (2019). Evaluation of polydentate picolinic acid chelating ligands and an α-melanocyte-stimulating hormone derivative for targeted alpha therapy using ISOL-produced 225Ac. EJNMMI Radiopharm Chem.

[CR24] Smith NA, Bowers DL, Ehst DA (2012). The production, separation, and use of 67Cu for radioimmunotherapy: a review. Appl Radiat Isot.

[CR25] Smith SV (2004). Molecular imaging with copper-64. J Inorg Biochem.

[CR26] Sprague JE, Peng Y, Fiamengo AL, Woodin KS, Southwick EA, Weisman GR (2007). Synthesis, characterization and in vivo studies of cu(II)-64-labeled cross-bridged tetraazamacrocycle-amide complexes as models of peptide conjugate imaging agents. J Med Chem.

[CR27] Spreckelmeyer S, Ramogida CF, Rousseau J, Arane K, Bratanovic I, Colpo N (2017). P-NO2-Bn-H4neunpa and H4neunpa-Trastuzumab: Bifunctional Chelator for Radiometalpharmaceuticals and 111In Immuno-single photon emission computed tomography imaging. Bioconjug Chem.

[CR28] Subat M, König B (2001). N-arylation of 1,4,7,10-tetraazacyclododecanes. Synthesis.

[CR29] Subat M, Woinaroschy K, Anthofer S, Malterer B, König B (2007). 1,4,7,10-tetraazacyclododecane metal complexes as potent promoters of carboxyester hydrolysis under physiological conditions. Inorg Chem.

[CR30] Tafreshi NK, Tichacek CJ, Pandya DN, Doligalski ML, Budzevich MM, Kil HJ (2019). Melanocortin 1 receptor–targeted a-particle therapy for metastatic uveal melanoma. J Nucl Med.

[CR31] Veiga AX, Arenz S, Erdélyi M (2013). N-Arylation of protected azamacrocycles. Synthesis.

[CR32] Vogenberg FR, Barash CI, Pursel M (2010). Personalized medicine - part 1: evolution and development into theranostics. Pharm Ther.

[CR33] Wadas TJ, Anderson CJ (2007). Radiolabeling of TETA- and CB-TE2A-conjugated peptides with copper-64. Nat Protoc.

[CR34] Yang H, Zhang C, Yuan Z, Rodriguez-Rodriguez C, Robertson A, Radchenko V (2020). Synthesis and evaluation of a new Macrocyclic Actinium-225 Chelator, quality control and in vivo evaluation of 225Ac-crown-αMSH peptide. Chemistry.

[CR35] Yordanova A, Eppard E, Kürpig S, Bundschuh RA, Schönberger S, Gonzalez-Carmona M (2017). Theranostics in nuclear medicine practice. Onco Targets Ther.

[CR36] Zhang C, Zhang Z, Lin KS, Lau J, Zeisler J, Colpo N (2018). Melanoma imaging using 18 F-labeled α-melanocyte-stimulating hormone derivatives with positron emission tomography. Mol Pharm.

[CR37] Zhang C, Zhang Z, Lin KS, Pan J, Dude I, Hundal-Jabal N (2017). Preclinical melanoma imaging with 68Ga-labeled α-melanocyte-stimulating hormone derivatives using PET. Theranostics.

[CR38] Zhang C, Zhang Z, Merkens H, Zeisler J, Colpo N, Hundal-Jabal N (2019). 18F-labeled cyclized α-melanocyte-stimulating hormone derivatives for imaging human melanoma Xenograft with positron emission tomography. Sci Rep.

[CR39] Zhang C, Zhang Z, Zeisler J, Colpo N, Lin K-S, Bénard F (2020). Selective Cycllized α-Melanocyte-Stimulating Hormone Derivative with Multiple N-Methylations for Melanoma Imaging with Positron Emission Tomography. ACS Omega..

