# Supplementary material for: Synthesis of DOTA-pyridine chelates for 64Cu coordination and radiolabeling of αMSH peptide
Source: EJNMMI Radiopharm Chem. 2021 Jan 13;6:3. doi: 10.1186/s41181-020-00119-4 (PMC7803858; doi:10.1186/s41181-020-00119-4)

Supporting Information

Synthesis of DOTA-pyridine chelates for ^{64}Cu coordination and radiolabeling of αMSH peptide

Hua Yang[1†], Feng Gao[1†], Brooke McNeil[1,2], Chengcheng Zhang[3], Zheliang Yuan[1], Stefan Zeisler[1], Joel Kumlin[1], Jutta Zeisler[3], François Bénard[3,4], Caterina Ramogida[1,2] and Paul Schaffer[1,2,4*]

[1]Life Sciences Division, TRIUMF, 4004 Wesbrook Mall, Vancouver, BC V6T 2A3, Canada.

[2]Department of Chemistry, Simon Fraser University, 8888 University Dr, Burnaby, BC V5A 1S6, Canada.

[3]Department of Molecular Oncology, BC Cancer Research Centre, 675 West 10th Ave, Vancouver, BC V5Z 1L3, Canada.

[4]Department of Radiology, University of British Columbia, 2775 Laurel St, Vancouver, BC V5Z 1M9, Canada.

[†]Both authors contributed equally to the study

[*]Correspondence: pschaffer@triumf.ca

IR spectra of DOTA-xPy (x=1-3) and Cu-DOTA-xPy (x=1-3)

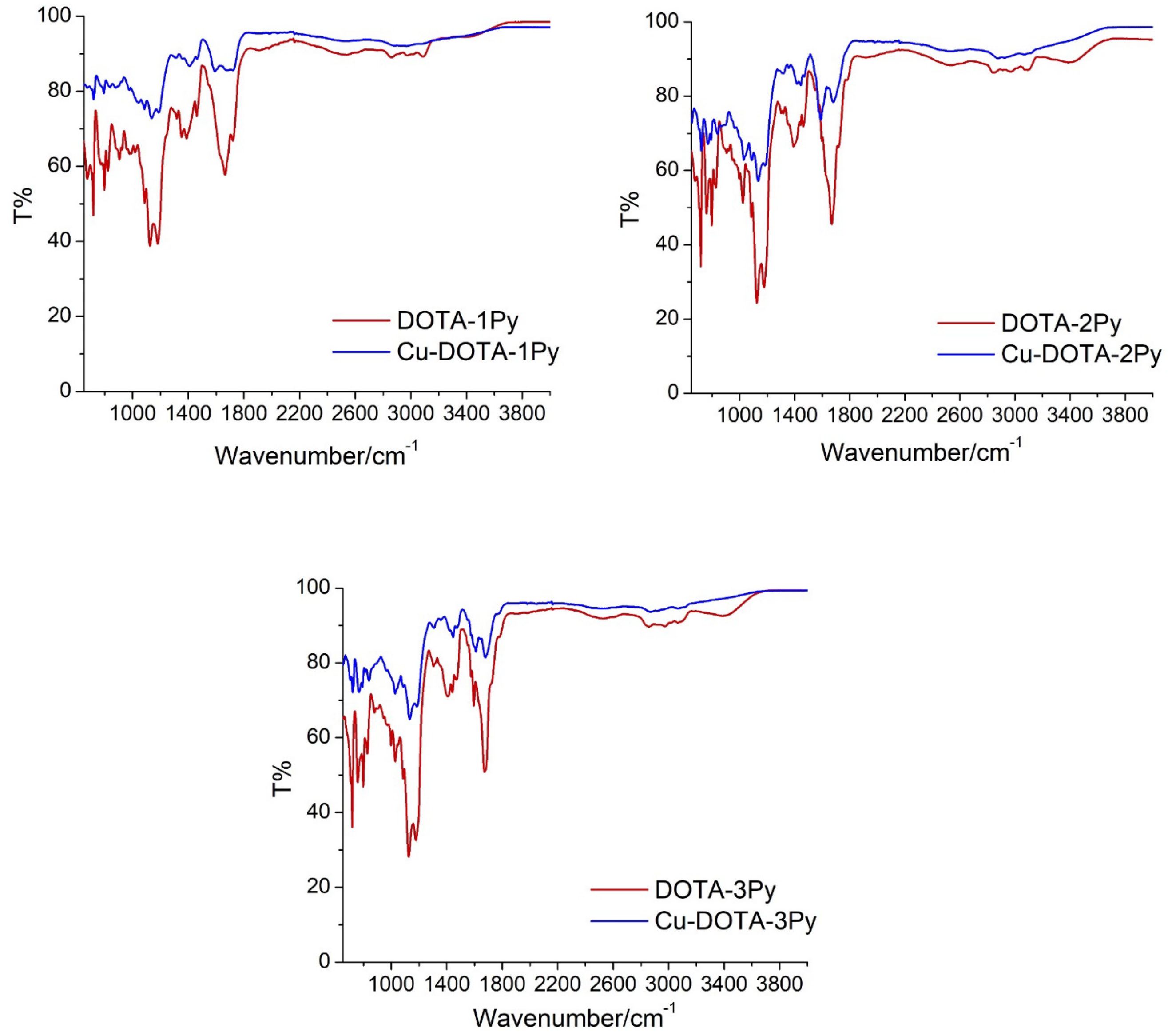

Fig. S1. IR spectra of DOTA-xPy (TFA salt, x=1-3) and Cu-DOTA-1Py (x=1-3).

UV-vis titration of DOTA-xPy (x=1-3) with $CuSO_4$

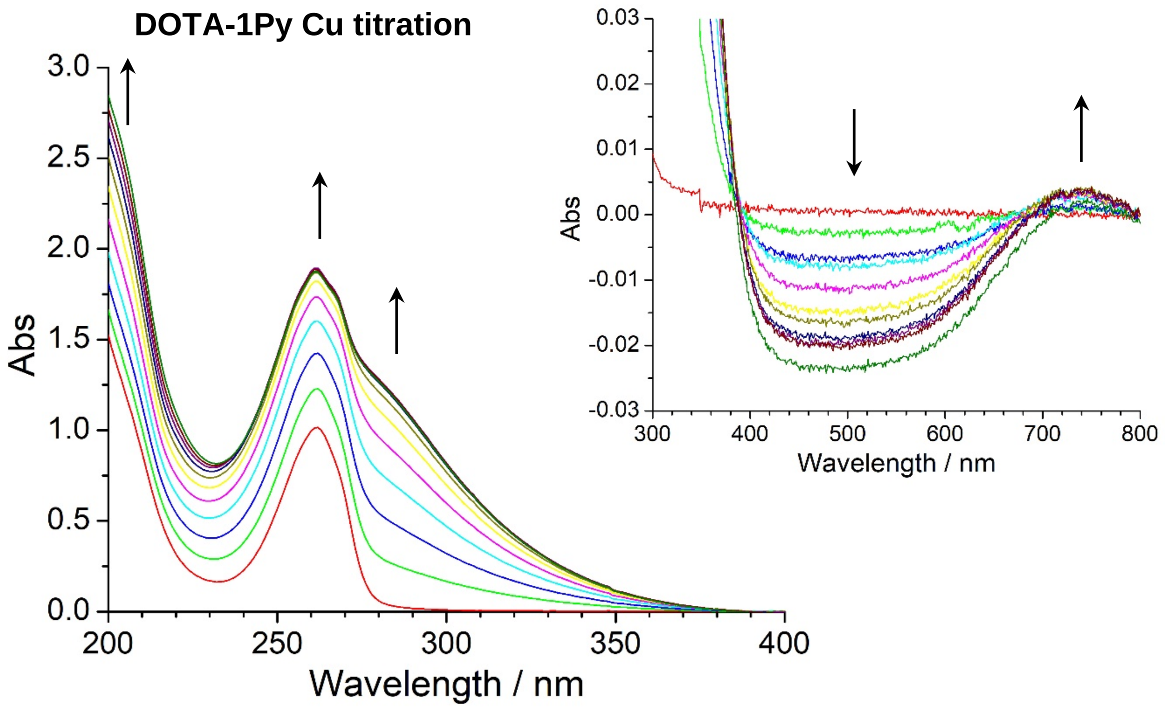
DOTA-1Py Cu titration
Abs
Wavelength / nm
3.0
2.5
2.0
1.5
1.0
0.5
0.0
200
250
300
350
400
0.03
0.02
0.01
0.00
-0.01
-0.02
-0.03
500
600
700
800

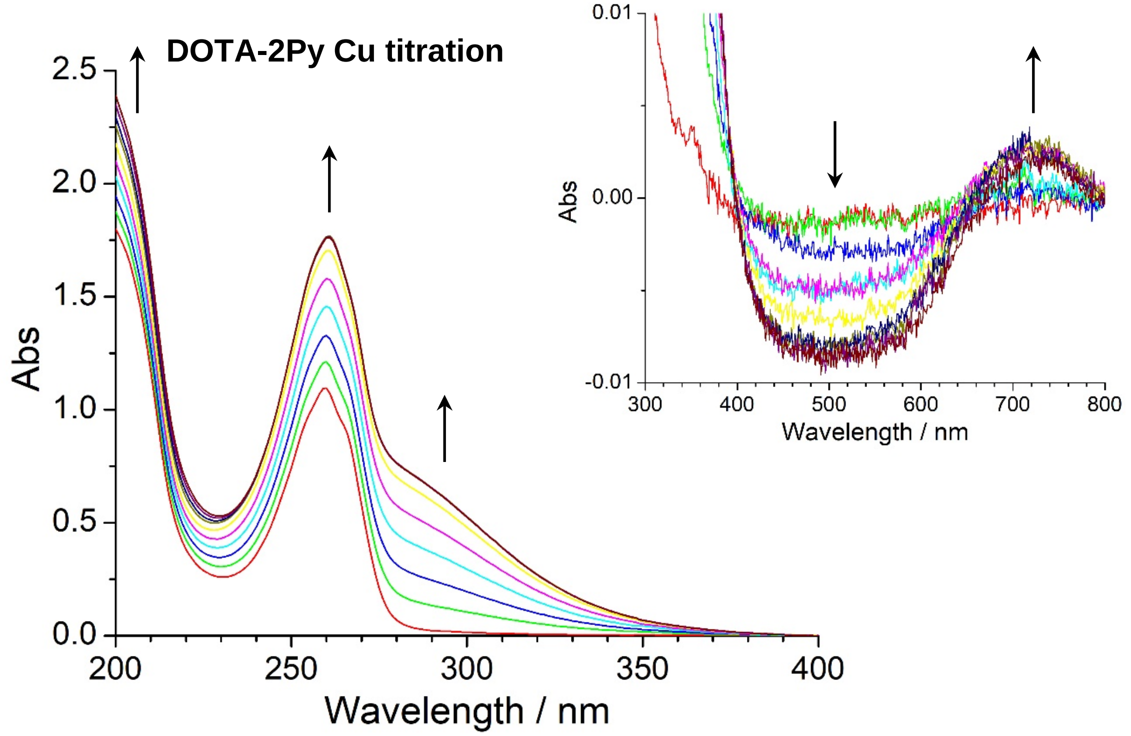
DOTA-2Py Cu titration
Abs
Wavelength / nm
2.5
2.0
1.5
1.0
0.5
0.0
200
250
300
350
400
0.01
0.00
-0.01
500
600
700
800

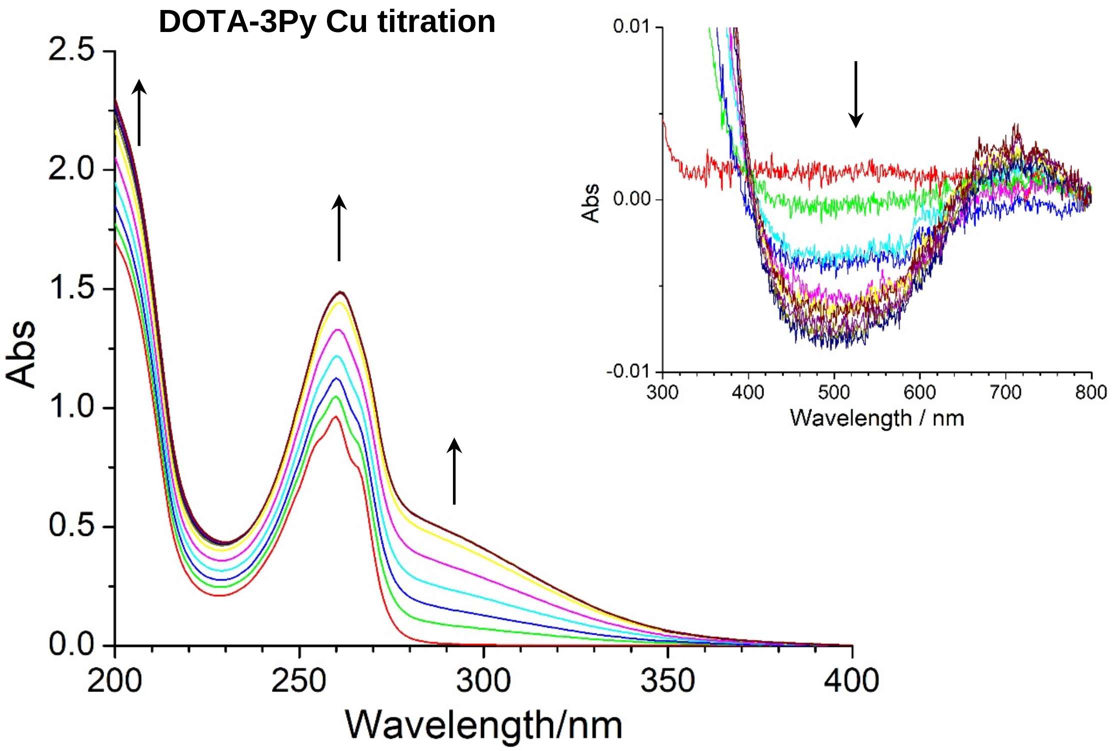

Fig. S2. UV-vis monitored titration of DOTA-xPy (x=1-3) with $CuSO_4$, with 0.2 eq Cu^{2+} added for each measurement.

DOTA-xPy (x=1-4) Concentration dependant ^{64}Cu labeling

Table S1. ^{64}Cu labeling yield at various ligand concentrations for DOTA, DOTA-1Py, DOTA-2Py, DOTA-3Py, and DOTA-4Py (n=3)

Log([L]/M)	^{64}Cu-DOTA-1Py	^{64}Cu-DOTA-2Py	^{64}Cu-DOTA-3Py	^{64}Cu-DOTA-4Py	^{64}Cu-DOTA
-3	68.1 ± 1.28	100	100	100	3.3 ± 0.29
-4	77.7 ± 0.93	100	100	100	3.3 ± 0.35
-5	48.5 ± 1.17	79.7 ± 1.47	94.5 ± 2.86	80.7 ± 2.14	2.8 ± 0.12
-6	3.9 ± 0.21	10.1 ± 0.59	8.8 ± 0.71	8.2 ± 0.52	0
-7	0	1.8 ± 0.17	3.1 ± 0.14	0	0
-8	0	0	0	0	0

^{64}Cu-DOTA-2Py-COOH-αMSH HPLC under isocratic conditions

^{64}Cu-DOTA-2Py-COOH-αMSH was produced using the same method as described above. The HPLC was run under method: Phenomenex Synergi Polar-RP column (4.6 mm x 250 mm), 1ml/min, isocratic 77% H_2O, 23% CH_3CN, 0.1% TFA. Gamma trace suggested the presence of isomers.

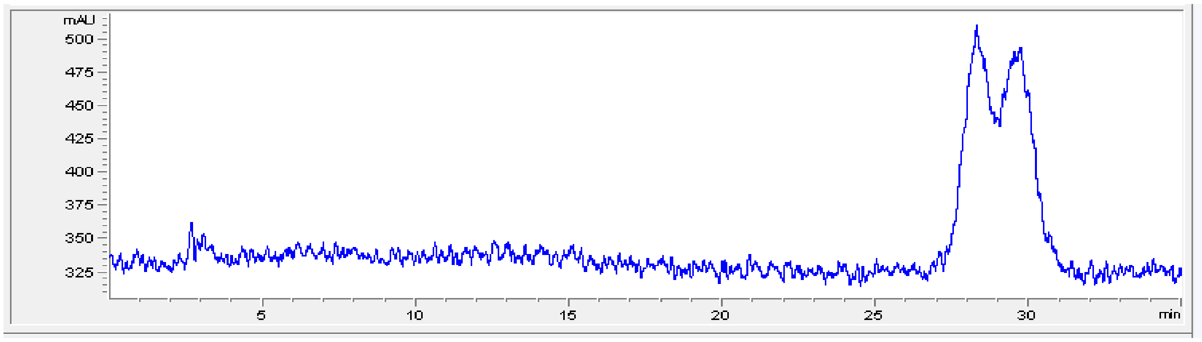

Fig. S3. HPLC gamma trace of ^{64}Cu-DOTA-2Py-COOH-αMSH in isocratic condition.

NMR Spectra

Cyclen-1Py

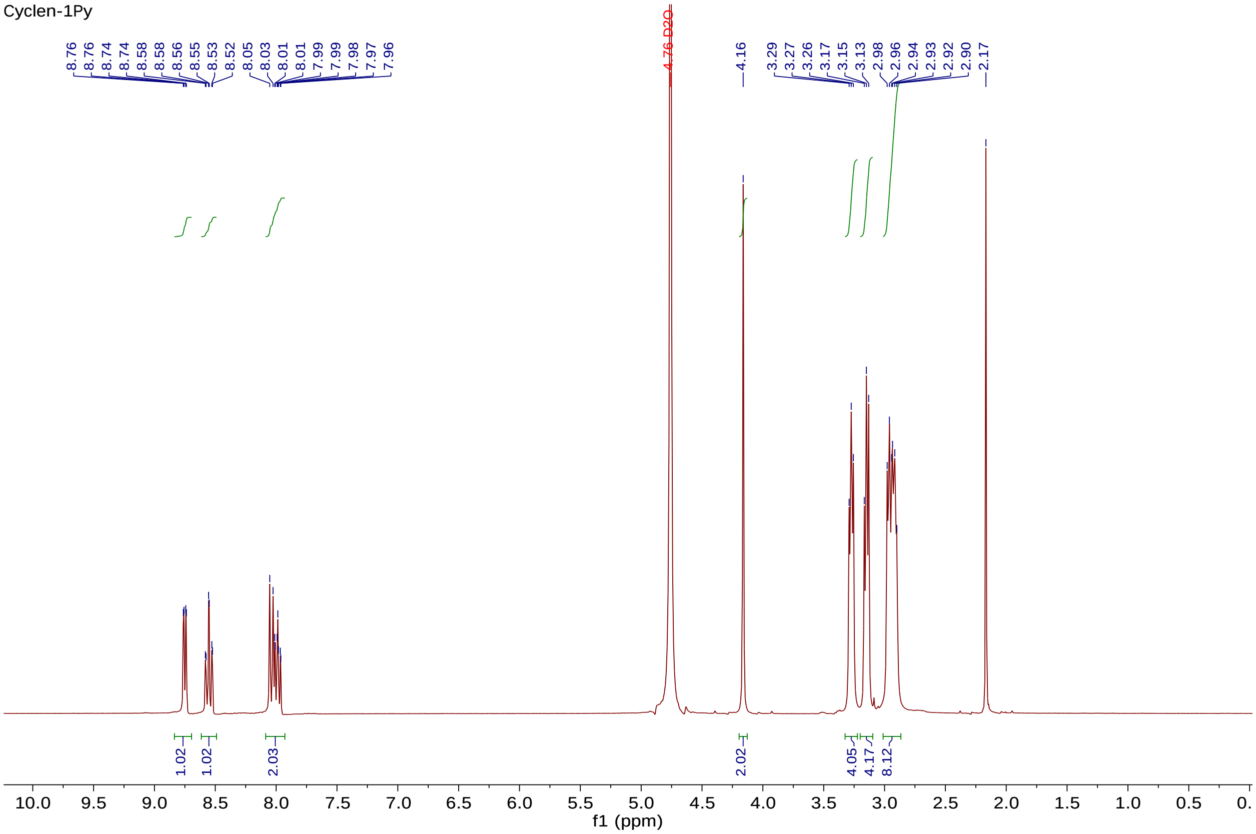

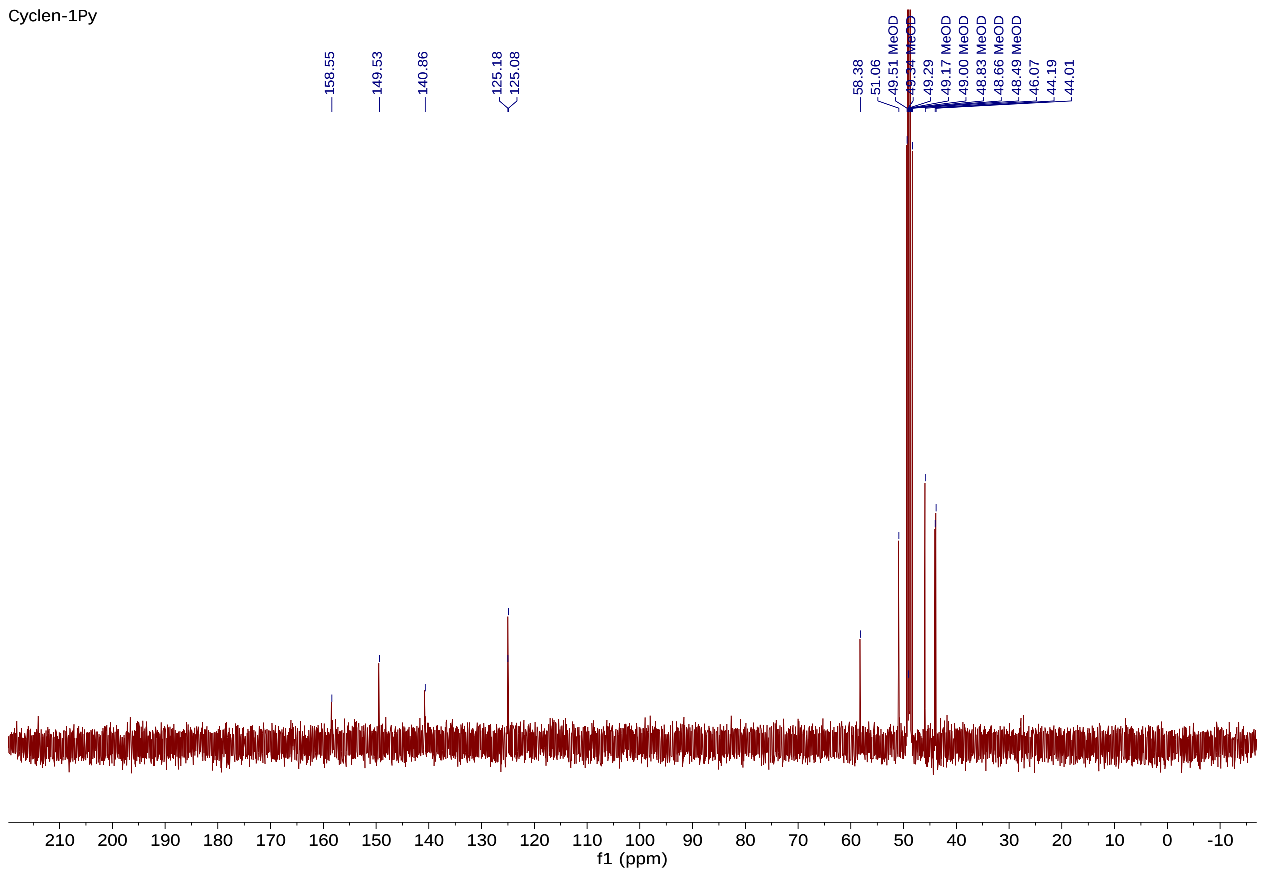
Cyclen-1Py
158.55
149.53
140.86
125.18
125.08
58.38
51.06
49.51 MeOD
49.34 MeOD
49.29
49.17 MeOD
49.00 MeOD
48.83 MeOD
48.66 MeOD
48.49 MeOD
46.07
44.19
44.01
210 200 190 180 170 160 150 140 130 120 110 100 90 80 70 60 50 40 30 20 10 0 -10
f1 (ppm)

Cyclen-2Py

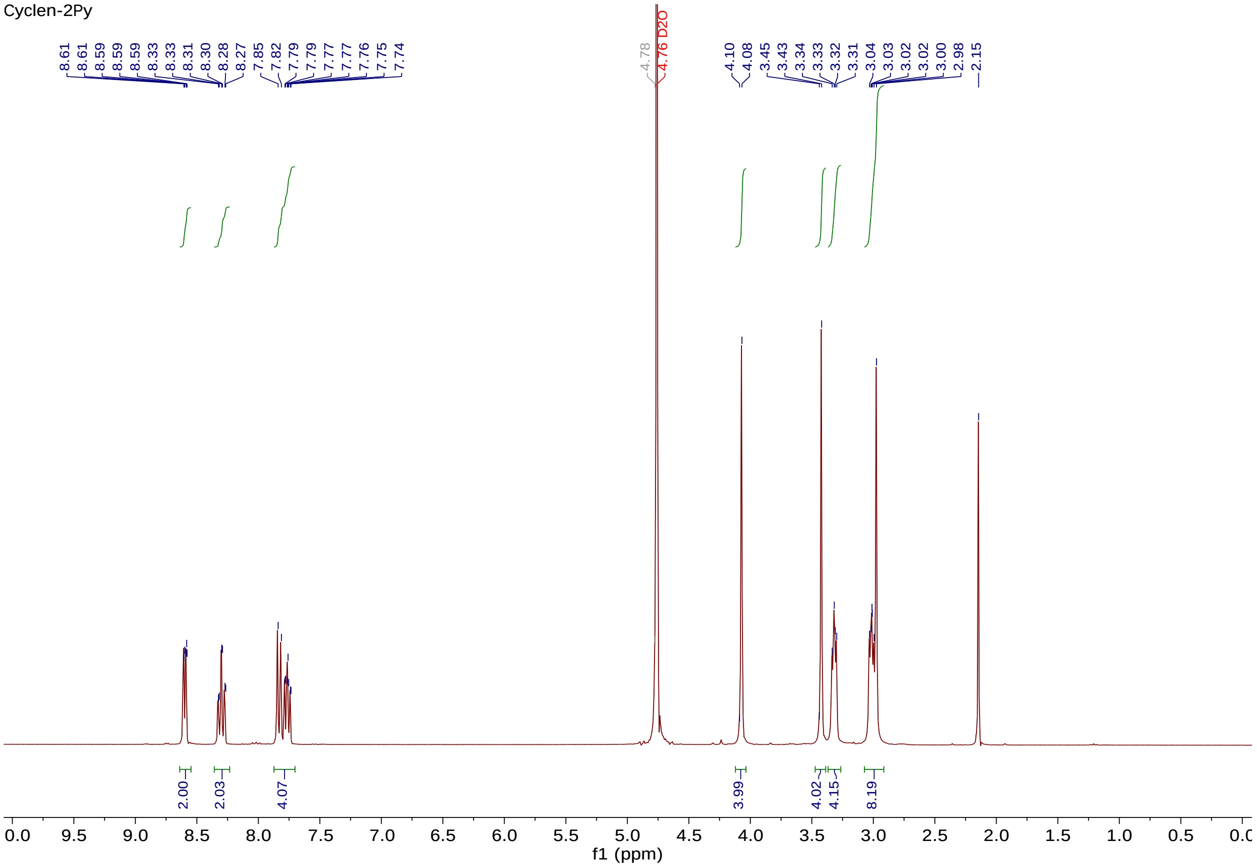
8.61
8.61
8.59
8.59
8.59
8.33
8.31
8.30
8.28
8.27
7.85
7.82
7.79
7.79
7.77
7.77
7.76
7.75
7.74
4.78
4.76 D2O
4.10
4.08
3.45
3.43
3.34
3.33
3.32
3.31
3.04
3.03
3.02
3.02
3.00
2.98
2.15
2.00
2.03
4.07
3.99
4.02
4.15
8.19
0.0
9.5
9.0
8.5
8.0
7.5
7.0
6.5
6.0
5.5
5.0
4.5
4.0
3.5
3.0
2.5
2.0
1.5
1.0
0.5
0.0
f1 (ppm)

Cylen-2Py

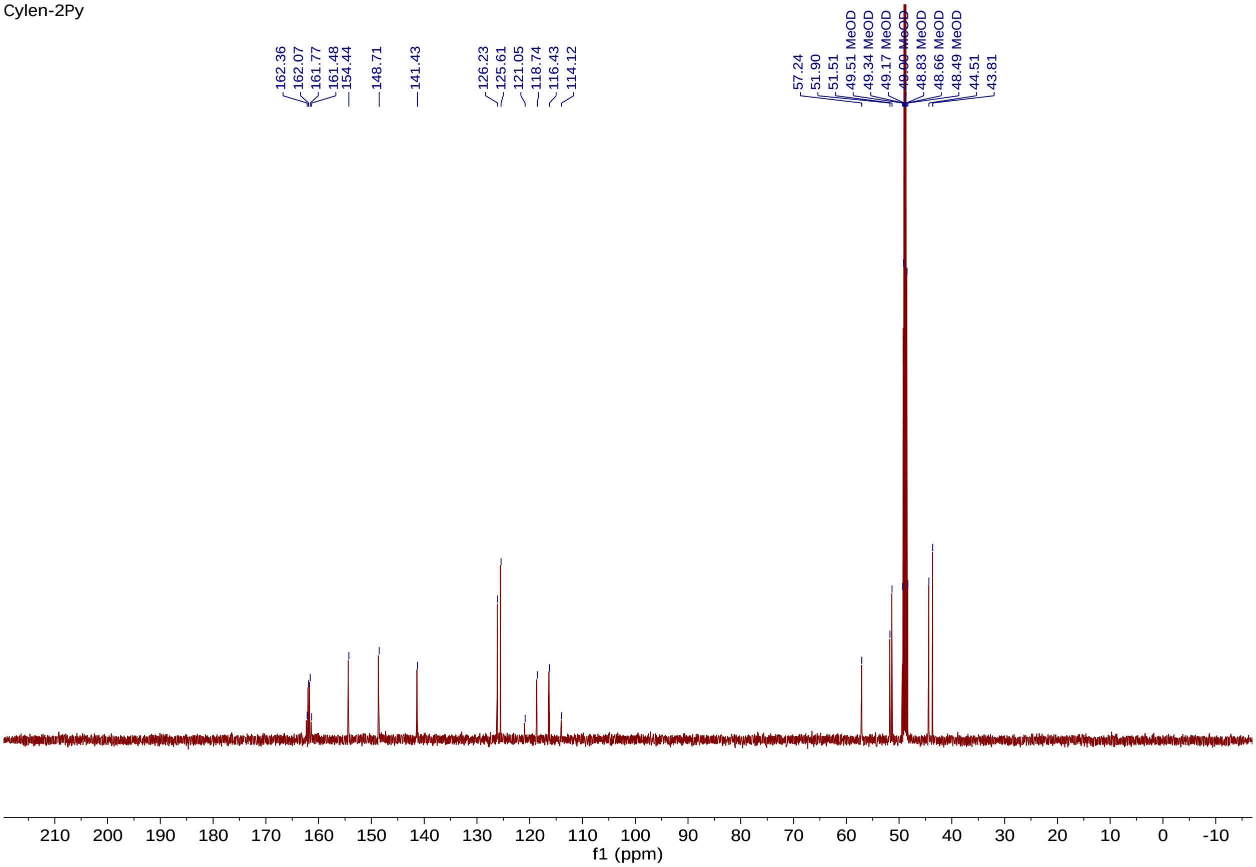
162.36
162.07
161.77
161.48
154.44
148.71
141.43
126.23
125.61
121.05
118.74
116.43
114.12
57.24
51.90
51.51
49.51 MeOD
49.34 MeOD
49.17 MeOD
49.00 MeOD
48.83 MeOD
48.66 MeOD
48.49 MeOD
44.51
43.81
210 200 190 180 170 160 150 140 130 120 110 100 90 80 70 60 50 40 30 20 10 0 -10
f1 (ppm)

Cyclen-3Py

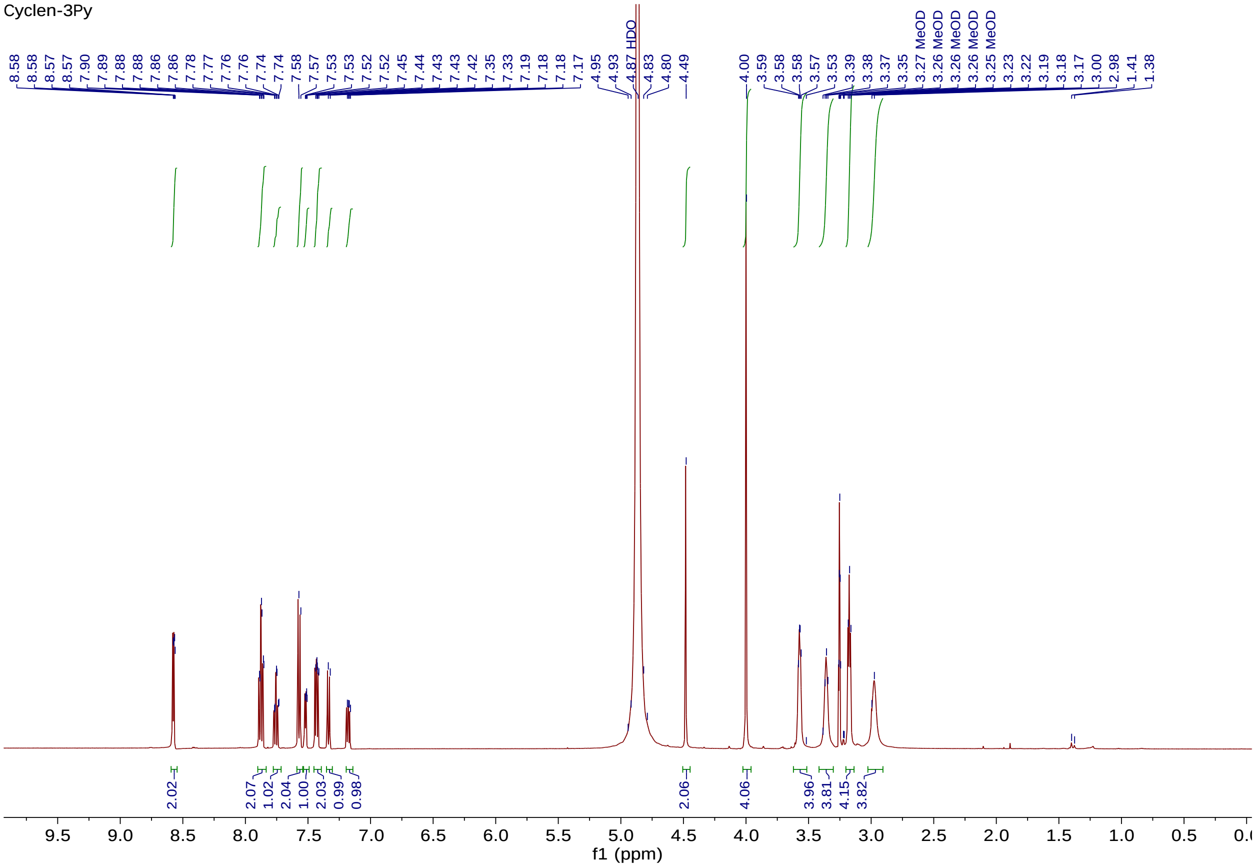

8.58
8.58
8.57
7.90
7.89
7.88
7.88
7.86
7.86
7.78
7.77
7.76
7.76
7.74
7.74
7.58
7.57
7.53
7.53
7.52
7.52
7.45
7.44
7.43
7.43
7.42
7.35
7.33
7.19
7.18
7.18
7.17
4.95
4.93
4.87 HDO
4.83
4.80
4.49
4.00
3.59
3.58
3.58
3.57
3.53
3.39
3.38
3.37
3.35
3.27 MeOD
3.26 MeOD
3.26 MeOD
3.26 MeOD
3.25 MeOD
3.23
3.22
3.19
3.18
3.17
3.00
2.98
1.41
1.38
2.02
2.07
1.02
2.04
1.00
2.03
0.99
0.98
2.06
4.06
3.96
3.81
4.15
3.82
9.5
9.0
8.5
8.0
7.5
7.0
6.5
6.0
5.5
5.0
4.5
4.0
3.5
3.0
2.5
2.0
1.5
1.0
0.5
f1 (ppm)

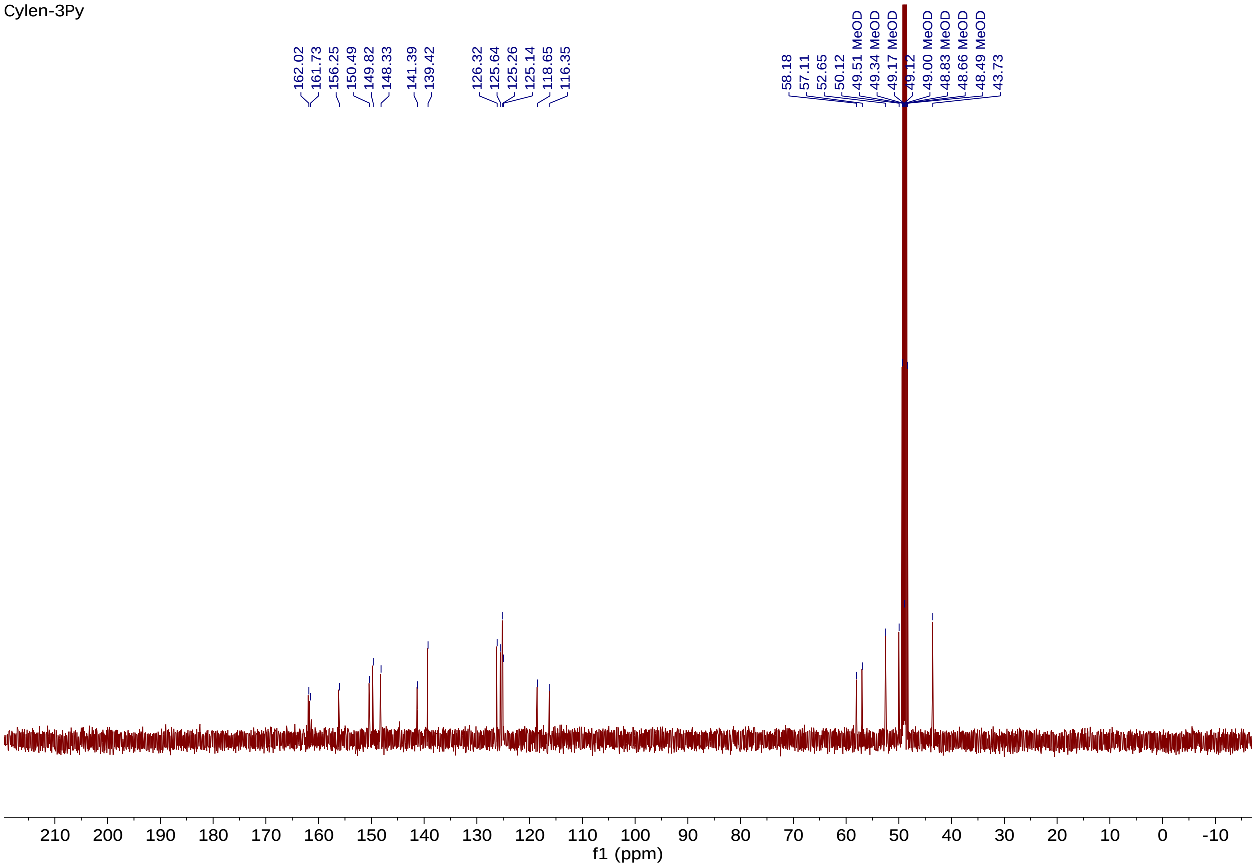
Cylen-3Py
162.02
161.73
156.25
150.49
149.82
148.33
141.39
139.42
126.32
125.64
125.26
125.14
118.65
116.35
58.18
57.11
52.65
50.12
49.51 MeOD
49.34 MeOD
49.17 MeOD
49.12
49.00 MeOD
48.83 MeOD
48.66 MeOD
48.49 MeOD
43.73
210 200 190 180 170 160 150 140 130 120 110 100 90 80 70 60 50 40 30 20 10 0 -10
f1 (ppm)

DOTA-2Py

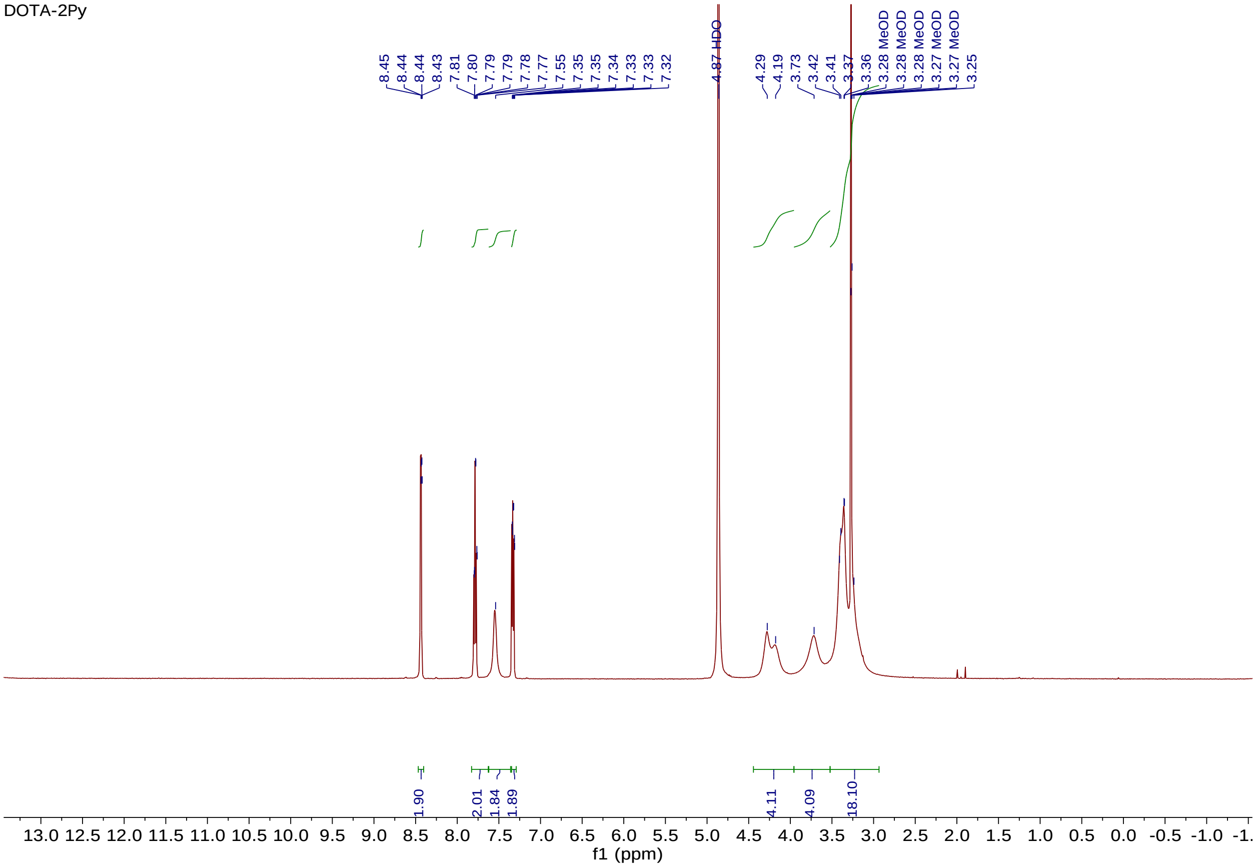
8.45
8.44
8.44
8.43
7.81
7.80
7.79
7.79
7.78
7.77
7.55
7.35
7.35
7.34
7.33
7.33
7.32
4.87 HDO
4.29
4.19
3.73
3.42
3.41
3.37
3.36
3.28 MeOD
3.28 MeOD
3.28 MeOD
3.27 MeOD
3.27 MeOD
3.25
1.90
2.01
1.84
1.89
4.11
4.09
18.10
13.0 12.5 12.0 11.5 11.0 10.5 10.0 9.5 9.0 8.5 8.0 7.5 7.0 6.5 6.0 5.5 5.0 4.5 4.0 3.5 3.0 2.5 2.0 1.5 1.0 0.5 0.0 -0.5 -1.0 -1.
f1 (ppm)

DOTA-2Py

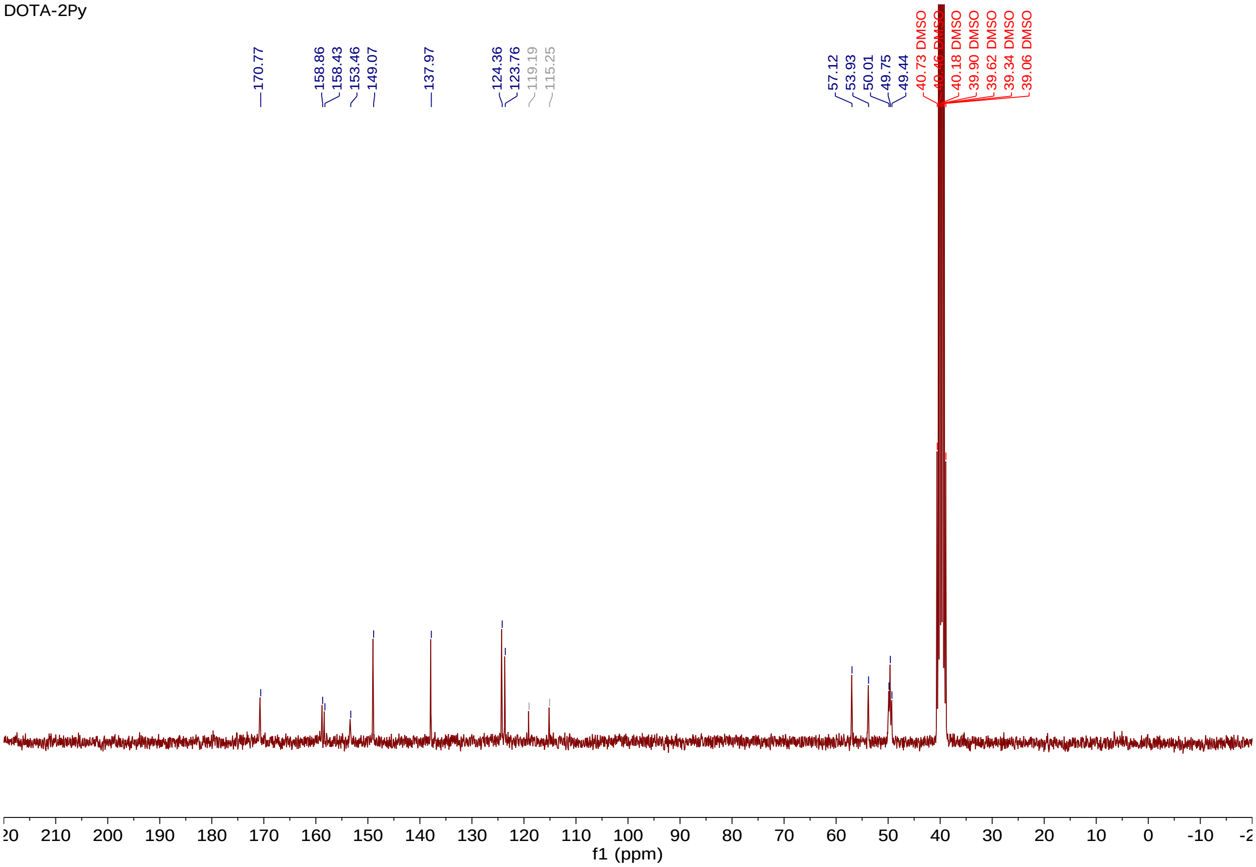
170.77
158.86
158.43
153.46
149.07
137.97
124.36
123.76
119.19
115.25
57.12
53.93
50.01
49.75
49.44
40.73 DMSO
40.18 DMSO
39.90 DMSO
39.62 DMSO
39.34 DMSO
39.06 DMSO
f1 (ppm)

DOTA-3Py

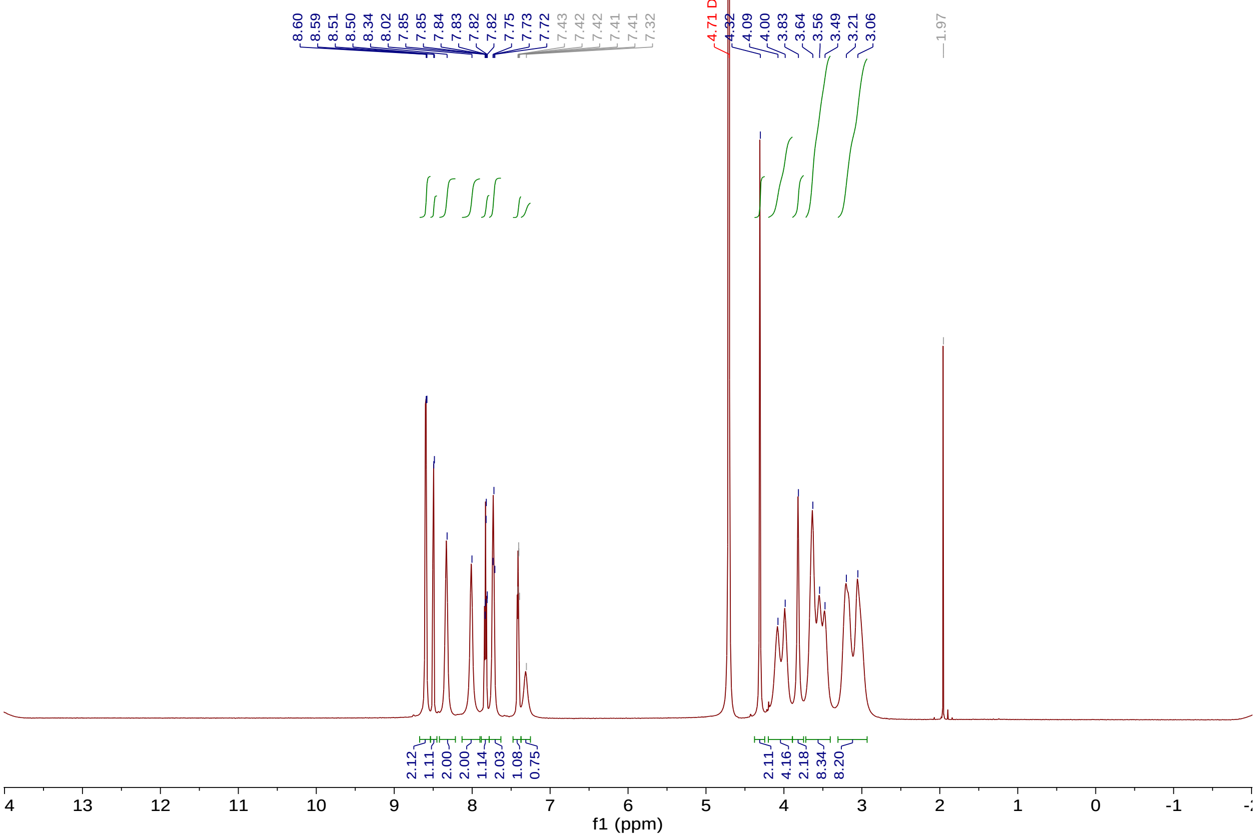

8.60
8.59
8.51
8.50
8.34
8.02
7.85
7.84
7.83
7.82
7.82
7.75
7.73
7.72
7.43
7.42
7.42
7.41
7.41
7.32
4.71 D2O
4.32
4.09
4.00
3.83
3.64
3.56
3.49
3.21
3.06
1.97
2.12
1.11
2.00
2.00
1.14
2.03
1.08
0.75
2.11
4.16
2.18
8.34
8.20
f1 (ppm)

DOTA-3Py

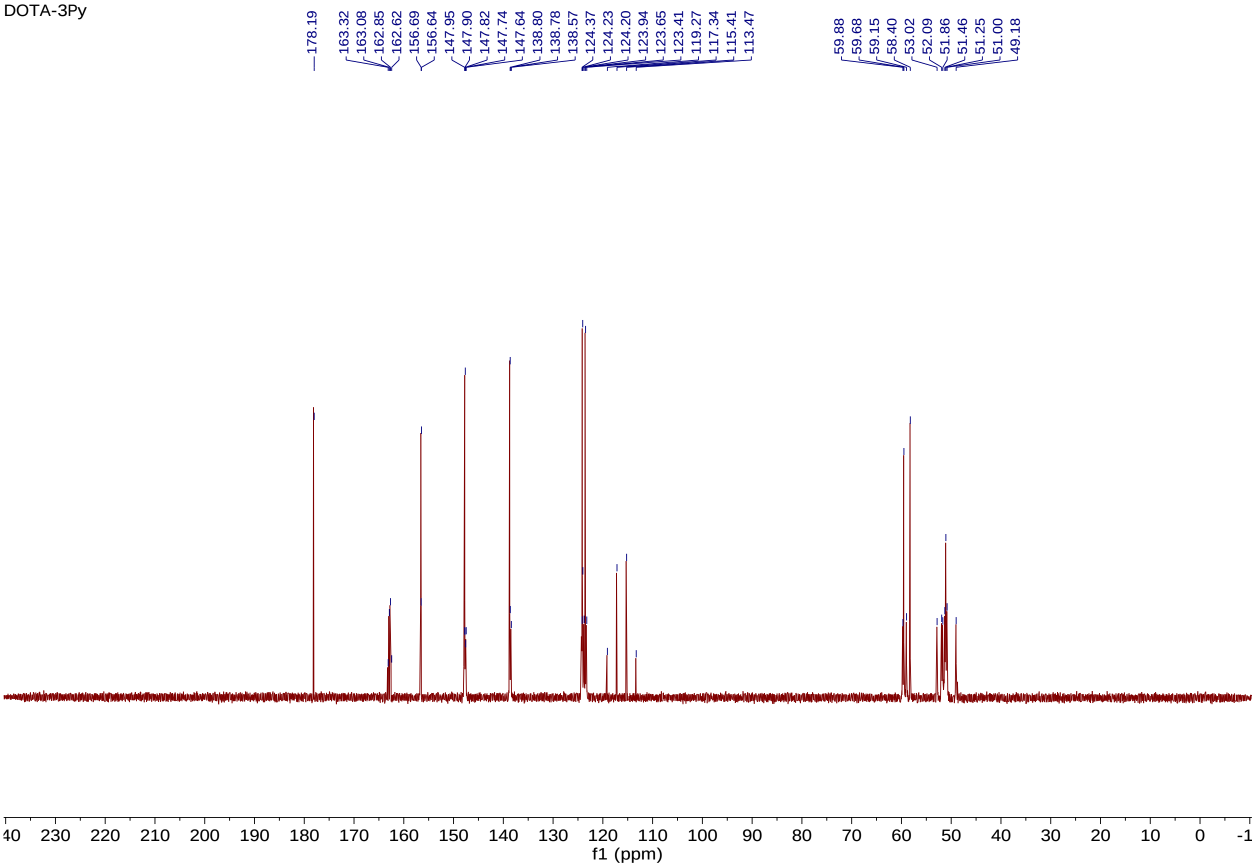
178.19
163.32
163.08
162.85
162.62
156.69
156.64
147.95
147.90
147.82
147.74
147.64
138.80
138.78
138.57
124.37
124.23
124.20
123.94
123.65
123.41
119.27
117.34
115.41
113.47
59.88
59.68
59.15
58.40
53.02
52.09
51.86
51.46
51.25
51.00
49.18
40 230 220 210 200 190 180 170 160 150 140 130 120 110 100 90 80 70 60 50 40 30 20 10 0 -1
f1 (ppm)

Mass Spectroscopy

DOTA-1Py-αMSH

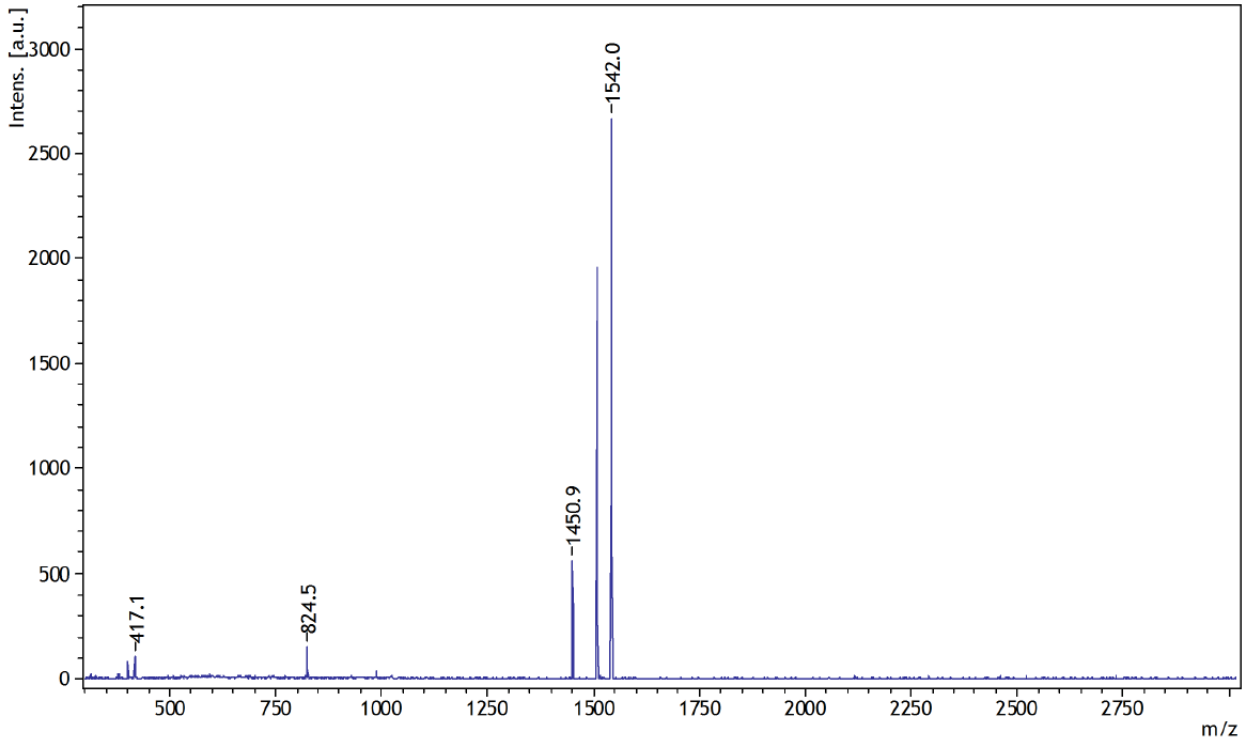

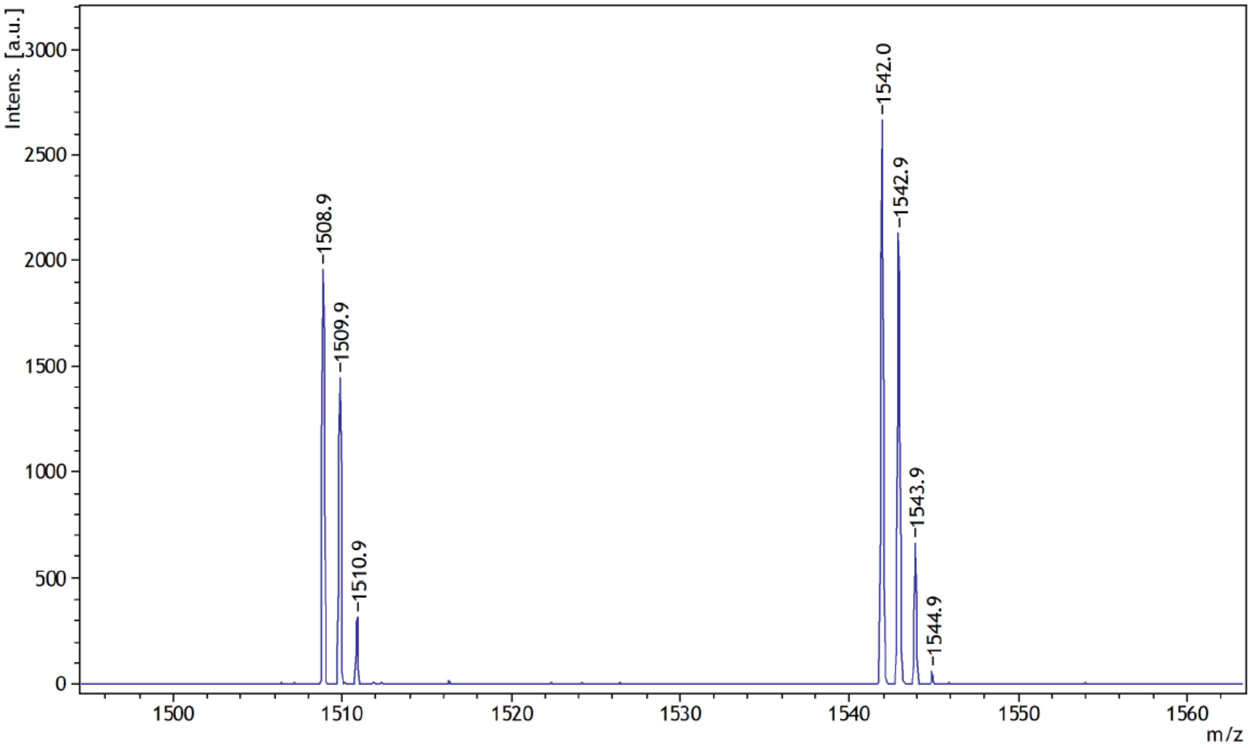

DOTA-2Py-αMSH

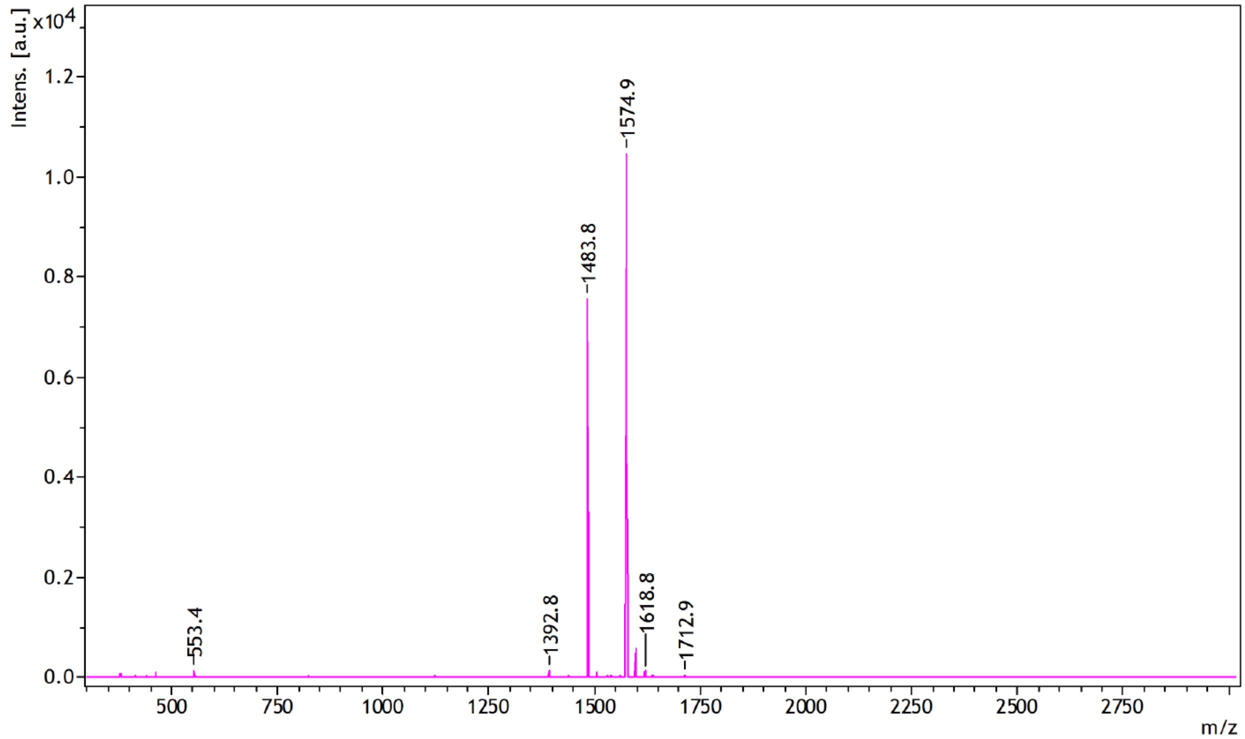

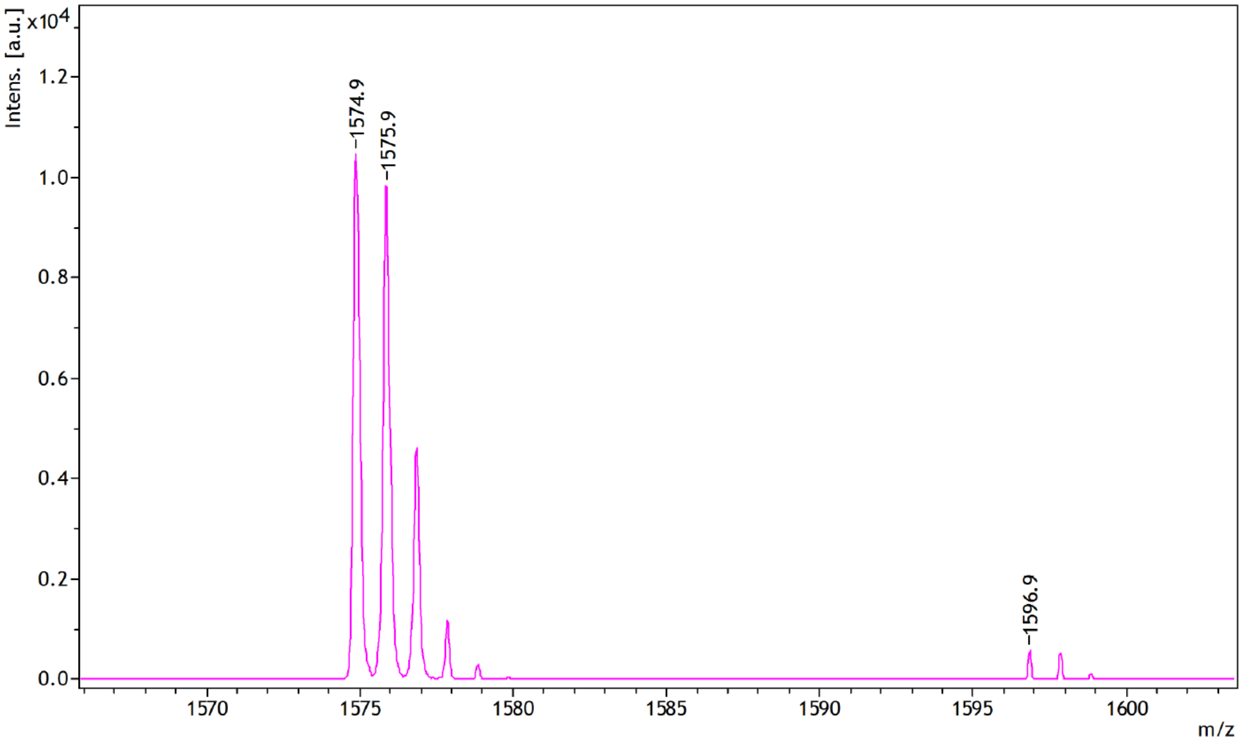

DOTA-3Py-αMSH

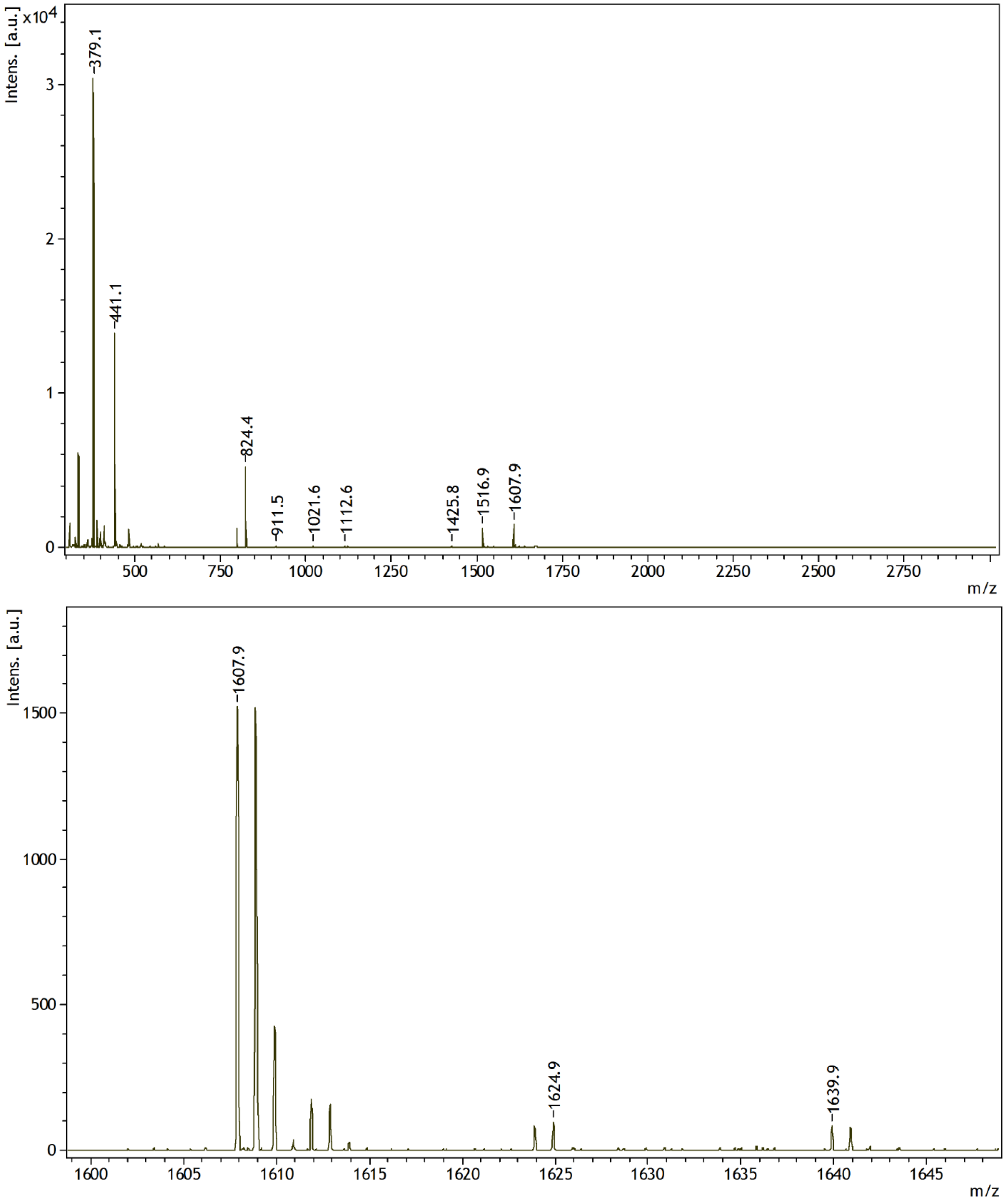

Cu-DOTA-1Py-αMSH

Spectrum RT 0.21 - 0.51 {11 scans} - Background Subtracted 0.03 - 0.15
2020-11-11 1Py-CycMSH Cu complex_1.datx 2020.11.11 14:10:34 ;
Intensity ESI + Max: 7E6

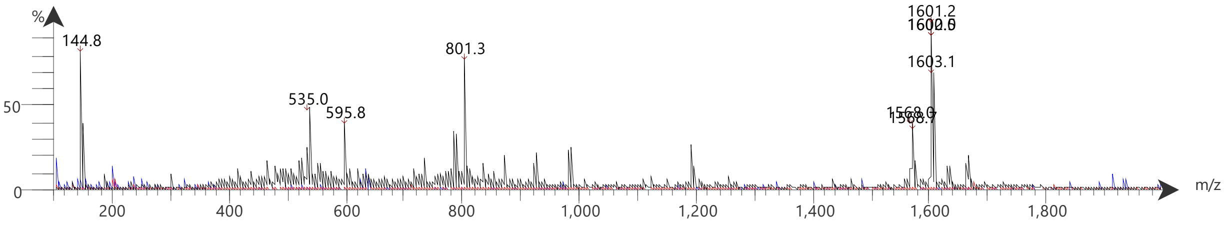

Cu-DOTA-2Py-αMSH

Spectrum RT 0.27 - 0.67 {14 scans} - Background Subtracted 0.03 - 0.15
2020-11-11 2Py-CycMSH Cu complex_1.datx 2020.11.11 14:12:39 ;
Intensity ESI + Max: 1.3E7

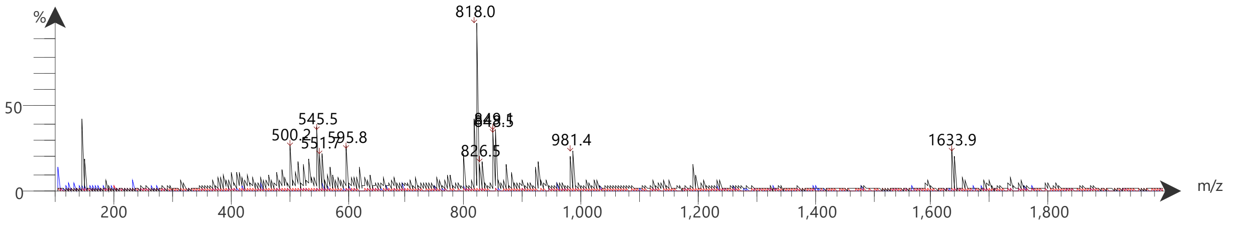

Cu-DOTA-3Py-αMSH

Spectrum RT 0.30 - 0.55 {9 scans} - Background Subtracted 0.00 - 0.15
2020-11-11 3Py-CycMSH Cu complex_1.datx 2020.11.11 14:17:38 ;
Intensity ESI + Max: 1.1E7

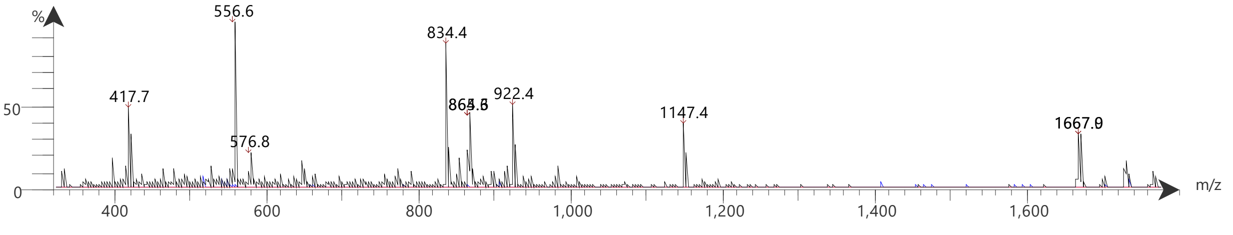

Supplement: Supplementary file 1 — Additional file 1. [file 41181_2020_119_MOESM1_ESM.pdf]
